# Single‐nucleus RNA sequencing identifies a novel tenogenic heterologous differentiation in endometrial carcinosarcomas: implications for diagnosis and tumor classification

**DOI:** 10.1002/path.70003

**Published:** 2026-01-15

**Authors:** Silvia González‐Martínez, José Palacios, Irene Carretero‐Barrio, Val Fernández‐Lanza, Alfonso Cortés‐Salgado, Javier Román, Xavier Matias‐Guiu, Sonia Gatius, Javier Cortés, Belén Pérez‐Mies

**Affiliations:** ^1^ ‘Contigo Contra el Cáncer de la Mujer’ Foundation Madrid Spain; ^2^ Molecular Pathology of Cancer Group, Ramón y Cajal Health Research Institute (IRYCIS) Madrid Spain; ^3^ Centre for Biomedical Research in Cancer Networks (CIBERONC), Carlos III Health Institute Madrid Spain; ^4^ Department of Pathology Ramón y Cajal University Hospital Madrid Spain; ^5^ Faculty of Medicine University of Alcalá Madrid Spain; ^6^ Centre for Biomedical Research in Infectious Diseases Networks (CIBERINFEC) Carlos III Health Institute Madrid Spain; ^7^ UCA‐GTB Unit, IRYCIS Madrid Spain; ^8^ Department of Medical Oncology Ramón y Cajal University Hospital, Ramón y Cajal Health Research Institute (IRYCIS) Madrid Spain; ^9^ IOB Madrid, Institute of Oncology Beata María Ana Hospital Madrid Spain; ^10^ Department of Medicine, Faculty of Biomedical and Health Sciences European University of Madrid Madrid Spain; ^11^ Department of Pathology, Hospital Universitari Arnau de Vilanova University of Lleida, IRBLLEIDA, CIBERONC Lleida Spain; ^12^ International Breast Cancer Center (IBCC), Pangaea Oncology Quiron Group Barcelona Spain; ^13^ Oncology Department Torrejón University Hospital, Ribera Group Madrid Spain; ^14^ Medica Scientia Innovation Research (MEDSIR) Barcelona Spain; ^15^ Medica Scientia Innovation Research (MEDSIR) Ridgewood NJ USA

**Keywords:** carcinosarcomas, endometrium, snRNA‐seq, plasticity, mesenchymal lineages, cellular heterogeneity, heterologous differentiation, tenogenic differentiation

## Abstract

Carcinosarcomas (CSs) are aggressive biphasic tumors characterized by epithelial and mesenchymal components, whose histogenesis and differentiation dynamics remain poorly understood. We present single‐nucleus RNA sequencing (snRNA‐seq) analysis of six CSs (five endometrial and one ovarian) and two normal endometrial samples, profiling over 96,298 cells. By integrating transcriptomic data with inferred copy number variations (CNVs), immunohistochemistry (IHC), fluorescence *in situ* hybridization (FISH), and *in situ* hybridization (ISH) validation, we resolved the complex cellular architecture of these tumors, identified lineage‐specific programs, and revealed unexpected differentiation trajectories. snRNA‐seq was used to further refine the histopathological classification of three cases by uncovering heterologous differentiation not previously recognized: one rhabdomyogenic, one osteogenic, and, notably, one exhibiting a novel tenogenic program, defined by the expression of *SCX*, *MKX*, and *TNMD*. All CSs displayed a prominent mesenchymal compartment comprising both undifferentiated fibroblast‐like cells and distinct lineage committed populations, including rhabdomyoblasts (Rhab), tenoblasts (Teno), osteoblasts (Osteo), and chondroblasts (Chond). In some tumors, multiple mesenchymal identities co‐existed, and in others, differentiation gradients (e.g. immature versus mature rhabdomyoblasts) were observed. These patterns underscore the cellular plasticity and multilineage potential of the sarcomatous component. Furthermore, the expression of specialized interface markers (*COL22A1*, *NCAM1*, *ACAN*, *CHRNG*, *MUSK*) suggests that some tumors use structured developmental programs reminiscent of the muscle–tendon junction, enthesis, or neuromuscular junction. CNV analysis revealed tumor‐specific genomic alterations with clonal and subclonal patterns linked to differentiation state, which were validated by FISH. Altogether, this study demonstrates that CSs are not static biphasic tumors but rather complex ecosystems with extensive developmental plasticity. Our findings redefine their classification and support the use of single‐nucleus approaches to uncover hidden differentiation trajectories in highly heterogeneous cancers, including the discovery of a previously unreported tenogenic lineage. Our results challenge the diagnosis of homologous CS when only morphological criteria are applied. © 2026 The Author(s). *The Journal of Pathology* published by John Wiley & Sons Ltd on behalf of The Pathological Society of Great Britain and Ireland.

## Introduction

Carcinosarcoma (CS) is an aggressive biphasic neoplasm composed of high‐grade carcinomatous and sarcomatous components [1]. CSs are infrequent tumors accounting for 5% and 2% of all uterine and ovarian malignancies, respectively, and cause ~16% of all deaths due to malignancies of the uterine corpus [2, 3]. Both uterine and ovarian CSs share similar patient demographics, outcomes, histology, and pathogenesis, with frequent *TP53* mutations [4, 5]. Histologically, they are classified as homologous when the sarcomatous component has a nonspecific appearance, and heterologous when the sarcomatous component differentiates to tissues that are not normally present, such as rhabdomyosarcoma, chondrosarcoma, or osteosarcoma. Other differentiations, such as liposarcoma or angiosarcoma, are extremely infrequent [6, 7].

Heterologous elements are present in ~43% of uterine CSs, with rhabdomyosarcoma in ~18% of cases [8, 9], and are associated with a worse prognosis, at least for FIGO (International Federation of Gynecology and Obstetrics) stage I–II disease [10]. However, the current WHO guidelines do not recommend routine immunohistochemistry (IHC) to identify specific heterologous differentiation, and classification relies on morphology. Since CSs show a high degree of heterogeneity and the identification of focal heterologous differentiation can be difficult, the proportion of tumors with heterologous differentiation is probably underestimated.

Molecular studies have demonstrated that the carcinomatous and sarcomatous components are clonally related and share common genetic alterations, with *TP53* mutation being the most frequent. In addition, we previously reported that complete epithelial‐to‐mesenchymal transition (EMT) occurred in CSs and described some of the molecular drivers of this process, including the role of several miRNAs and transcription factors [11]. These observations were subsequently confirmed by other studies, including the TCGA cohort [12]. EMT is not a binary process or a single program, as it implies the generation of intermediate hybrid epithelial–mesenchymal states that are difficult to recognize using bulk RNA analysis. Single‐nucleus RNA sequencing (snRNA‐seq) has emerged as a robust tool for characterizing cell subpopulations and identifying cellular heterogeneity in normal and pathological tissues. Recently, several studies have demonstrated the suitability of snRNA‐seq analysis using formalin‐fixed paraffin‐embedded (FFPE) tissue to generate transcriptomic profiles and identify even minor subpopulations of cells within heterogeneous samples [13, 14].

Few studies have analyzed endometrial carcinomas using single‐cell (sc) RNA‐ or snRNA‐seq, and have included a low number of cases. In our recent review, only 29 samples were analyzed, mostly endometrioid (*n* = 19), with one serous carcinoma; the histological type was unspecified in nine tumors [15]. At present, no scRNA‐ or snRNA‐seq studies of endometrial CSs have been reported, although two ovarian CSs have been analyzed using this technology [16, 17].

In this exploratory and hypothesis‐generating study, we analyzed a series of endometrial CSs to characterize their cellular composition and transcriptional programs at single‐nucleus resolution, with a particular focus on EMT and mesenchymal differentiation. We also included one ovarian CS to explore potential site‐specific differences. Using FFPE‐derived snRNA‐seq, we aimed to identify distinct tumor cell populations, delineate lineage relationships, and uncover potential diagnostic and therapeutic targets associated with specific heterologous components. To our knowledge, this represents the most comprehensive single‐cell transcriptomic analysis of CSs to date.

## Materials and methods

### Sample collection

FFPE samples were collected from therapy‐naïve CS tissues obtained from six patients undergoing primary surgery and from two healthy donors. Histological sections were evaluated to identify and select the most suitable FFPE blocks for snRNA‐seq analysis. A summary of the clinicopathological characteristics of all patients and donors is provided in Table 1.

**Table 1:** Clinicopathological characteristics of patients and carcinosarcoma cases included in the study.

Feature	CS1	CS2	CS3	CS4	CS5	CS6	N1	N2
Histological diagnosis	Heterologous ECS with rhabdomyoblastic differentiation	Homologous ECS	Heterologous ECS with rhabdomyoblastic differentiation	Homologous ECS	Homologous ECS	Heterologous OCS with chondroid differentiation	Normal	Normal
Age (years)	58	73	57	68	82	74	49	50
Outcome	Alive and disease‐free	Alive and disease‐free	Alive with disease	Alive with disease	Deceased	Deceased		
Surgery	HT + adnexectomy + lymphadenectomy	HT + adnexectomy + lymphadenectomy	No surgery	HT + adnexectomy + omentectomy	HT + adnexectomy + omentectomy + lymphadenectomy	Adnexectomy + sigmoidectomy (stage IV, pleural metastases)		
LVI	No	No	NA	Yes, extensive (>4 spaces)	Yes (2 spaces)	NA		
Size (cm)	1.08	9	NA	8	4	NA		
pT	1a	1a	NA	3b	2	NA		
pN	0	0	NA	NA	0	NA		
% Carcinoma	15	10	30	10	15	40		
% Sarcoma	85	90	60	90	85	60		
ER	Negative	Focally positive	Negative	Negative	Focally positive	Focally positive		
PR	Negative	Focally positive	Negative	Negative	Focally positive	Focally positive		
MMR	Proficient	Proficient	Proficient	Proficient	Proficient	Proficient		
*POLE*	Negative	Technique failure	Negative	Negative	Negative	Negative		
p53 IHC pattern	Wild type	Null epithelial; cytoplasmic mesenquimal	Overexpression (both components)	Null (both components)	Overexpression (both components)	Overexpression (both components)		
Additional studies (NGS)	*TP53* c.920‐1G>A	*TP53* c.786del/*PIK3CA* V346G/*PIK3R1* Q432*/*PPP2R1A* P179R^†^	NA	NA	NA	*TP53* c.527G>A		
Molecular subtype	*TP53* mutated	*TP53* mutated	*TP53* mutated	*TP53* mutated	*TP53* mutated	*TP53* mutated		

^†^ NGS analysis was performed separately for the epithelial and mesenchymal components of the CS.

ECS, endometrial carcinosarcoma; HT, hysterectomy; MMR, mismatch repair proteins; NA, not available; OCS, ovarian carcinosarcoma.

### Ethics approval and patient consent

All procedures involving human participants followed institutional and national ethical guidelines and the Declaration of Helsinki [18]. Ethics approval was obtained from Ramón y Cajal University Hospital (ref. 226/19), and all participants provided written informed consent.

### Pathological and molecular characterization of carcinosarcomas

Tumor histological type and FIGO grade were evaluated by two expert pathologists, following the WHO fifth edition and FIGO criteria [19, 20]. We performed IHC studies on whole sections of primary tumors for surrogate molecular categorization. IHC results were analyzed following the 2023 ‘American Society of Clinical Oncology and the College of American Pathologists’ criteria [21]. A summary of the IHC panel is provided in supplementary material, Table S1. RNA ISH for tenomodulin and FISH for selected chromosomal alterations (*MYC*, *CCNE1*, *MDM4*) were also performed. Sequencing of DNA was carried out as previously reported [22] and was performed separately on epithelial and mesenchymal components where feasible. Details of the protocols and scoring criteria are available in the Supplementary materials and methods.

### Single‐nucleus RNA sequencing (snRNA‐seq)

FFPE samples were processed following the protocol CG000632, and single‐nucleus library preparation was conducted following the protocol CG000477 (10x Genomics, Pleasanton, CA, USA). The nuclei were loaded onto the Chromium X/iX for partitioning. Subsequently, the libraries were sequenced on a NovaSeq 6000 system (Illumina, San Diego, CA, USA).

### snRNA‐seq data processing

Reads were aligned to GRCh38 and processed with Cell Ranger (v7.1.0). Data were analyzed in R (v4.3.2) (https://cran.r-project.org/bin/windows/base/old/4.3.2/) using Seurat (v5.0.2) (https://github.com/satijalab/seurat/releases/tag/v5.0.2). Cells with more than 200 genes, log_10_(genes/UMI) > 0.8, and less than 20% mitochondrial reads were retained. Doublets were removed using doubletFinder (v2.0.4) [23]. Normalization used SCTransform(), followed by principal component analysis (PCA) and uniform manifold approximation and projection (UMAP). Clustering (resolution = 0.6) and subclustering (resolution = 0.2–0.5) were performed. Differential expression was identified using *FindAllMarkers*.

Putative CNV events were inferred as in our previous study [13], using normal epithelial cells as a reference baseline to estimate CNV profiles in malignant cells.

Further details on quality control metrics, bioinformatic parameters, and workflows are provided in supplementary material, Table S2 and Supplementary materials and methods.

### RT‐qPCR validation

Total RNA was extracted from FFPE tumor samples using the AllPrep DNA/RNA FFPE Kit (QIAGEN, Hilden, Germany). Complementary DNA (cDNA) was synthesized using the High‐Capacity cDNA Reverse Transcription Kit (Thermo Fisher Scientific, Waltham, MA, USA). Quantitative real‐time PCR (RT‐qPCR) was performed on a LightCycler 480 System (Roche, Basel, Switzerland) using SYBR Select Master Mix (Thermo Fisher Scientific). Predesigned gene‐specific primers were purchased from Integrated DNA Technologies (IDT, Coralville, IA, USA). Amplification reactions were carried out following the manufacturers’ protocols, and relative expression levels were calculated using the ΔΔCt method [24].

## Results

### Clinicopathological features of samples

**Figure 1:**
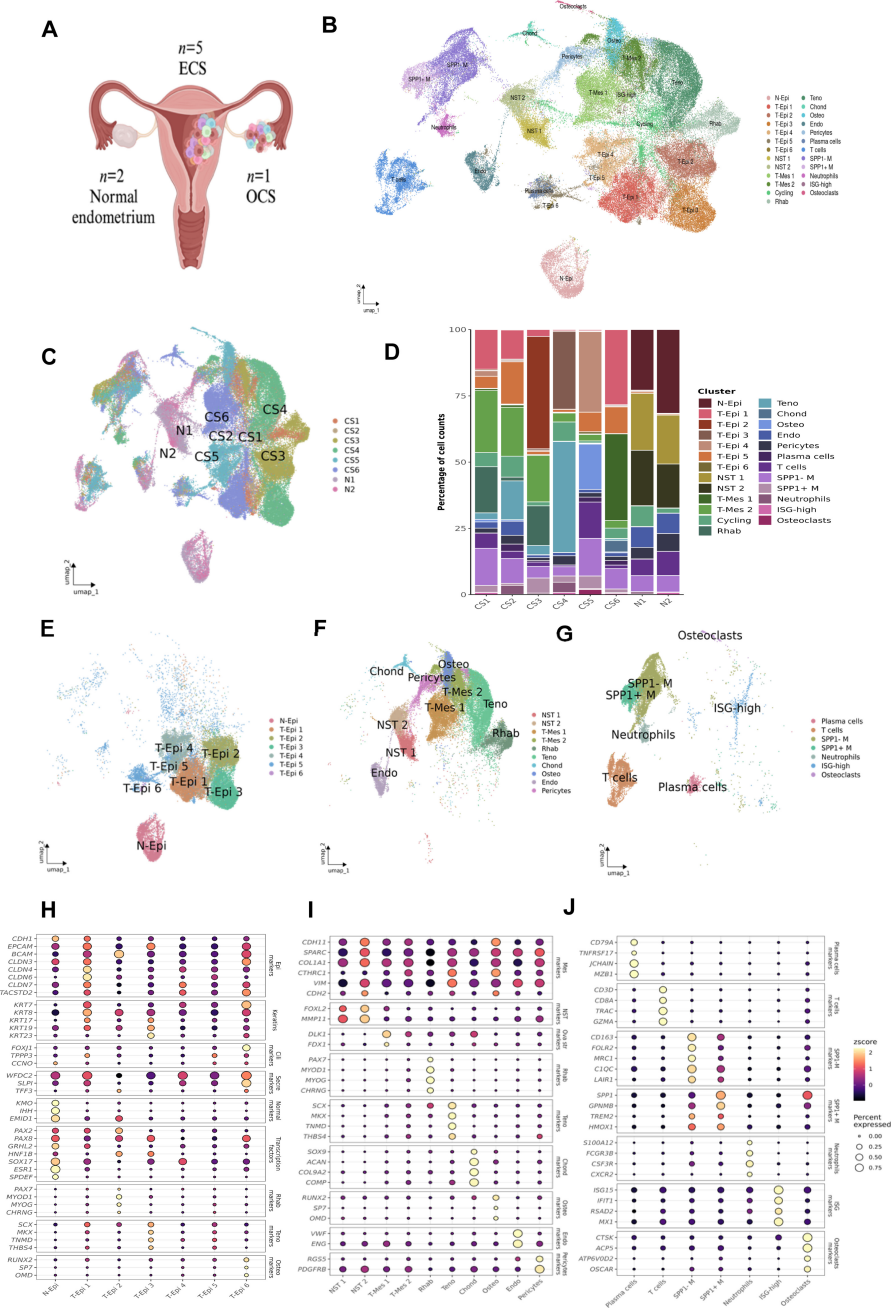
Single‐nucleus classification of carcinosarcomas. (A) Schematic representation of the female reproductive system showing the samples included in the study (created in BioRender.com; ID: VY28WHV332). (B) UMAP plot showing the clustering of single cells based on their transcriptomic profiles into 19 major cell types. (C) UMAP plot displaying the clustering of single cells, color‐coded by sample, to illustrate sample‐specific distribution. (D) Bar chart depicting the composition of cell types across different patients. (E–G) UMAP plots split by epithelial, stromal, and immune cells. (H–J) Dot plots illustrating the expression levels of marker genes for each identified cell type to confirm classification accuracy.

FFPE tissue samples from six CSs and two normal proliferative endometrium control samples were obtained from the Pathology Department of Ramón y Cajal University Hospital (Madrid, Spain). Clinicopathological details are summarized in Table 1 and Figure 1A.

Two tumors were initially diagnosed as heterologous CS with rhabdomyogenic differentiation (CS1, CS3), one as heterologous CS with chondrogenic differentiation (CS6), and three as homologous CS (CS2, CS4, CS5). However, snRNA‐seq challenged the initial classification in the three homologous cases, revealing that all CSs presented some form of heterologous differentiation with distinct cellular populations specific to each differentiation. Consequently, one homologous CS was reclassified as heterologous CS with rhabdomyogenic differentiation (CS2), one was determined to have tenogenic differentiation (CS4), and the other was found to exhibit osteogenic differentiation (CS5). The reclassification was confirmed by identifying the specific mesenchymal populations by IHC for myogenin and MyoD1 in CS2, for SATB2 in CS5, and by ISH for *TNMD* in CS4. These findings underscore the potential of snRNA‐seq to refine tumor classification based on molecular and cellular profiles.

### Single‐nucleus expression of normal proliferative endometrium and carcinosarcomas

We performed a comprehensive analysis, including quality control and dimensionality reduction, on snRNA‐seq data from 96,298 cells across the eight samples (Figure 1B,C). Based on classical cell‐type markers, we classified the cells into 25 major categories: normal epithelial cells (N‐Epi), normal stromal cells (NST 1–2), tumoral epithelial cells (T‐Epi 1–6), cycling cells (Cycling) (see supplementary material, Figure S1 for a detailed sub‐analysis), and malignant cells with mesenchymal (sarcomatous) differentiation (T‐Mes 1–2), which also included specific cell types such as rhabdomyoblasts (Rhab), tenoblasts (Teno), osteoblasts (Osteo), and chondroblasts (Chond). Additionally, endothelial cells (Endo), perycites, plasma cells, T cells, macrophages (SPP1^−^ M and SPP1^+^ M), neutrophils, interferon‐stimulated gene (ISG) high cells, and osteoclasts were identified (Figure 1B and supplementary material, Table S3). We then analyzed the distribution of these cell types across samples (Figure 1D and supplementary material, Table S3) and validated the classification accuracy by assessing the expression profiles of marker genes for each cell type (Figure 1E–J and supplementary material, Table S4).

### Normal proliferative endometrium

The normal endometrium was characterized by the expression of canonical epithelial markers such as *CDH1*, *ESR1*, *SOX17*, or *SPDEF*. Additionally, the epithelial cells expressed genes associated with the proliferative phase, including *KMO*, *IHH*, and *EMID1*, consistent with previous studies [25]. In the stromal compartment, NST 1, NST 2, and cycling cells were identified in both samples.

### Carcinosarcoma neoplastic cells

In all CSs, neoplastic cells with epithelial or mesenchymal differentiation were observed. The neoplastic nature of CS cells was evident due to the presence of CNVs (see below).

### Comparative gene expression between epithelial and sarcomatous compartments

**Figure 2:**
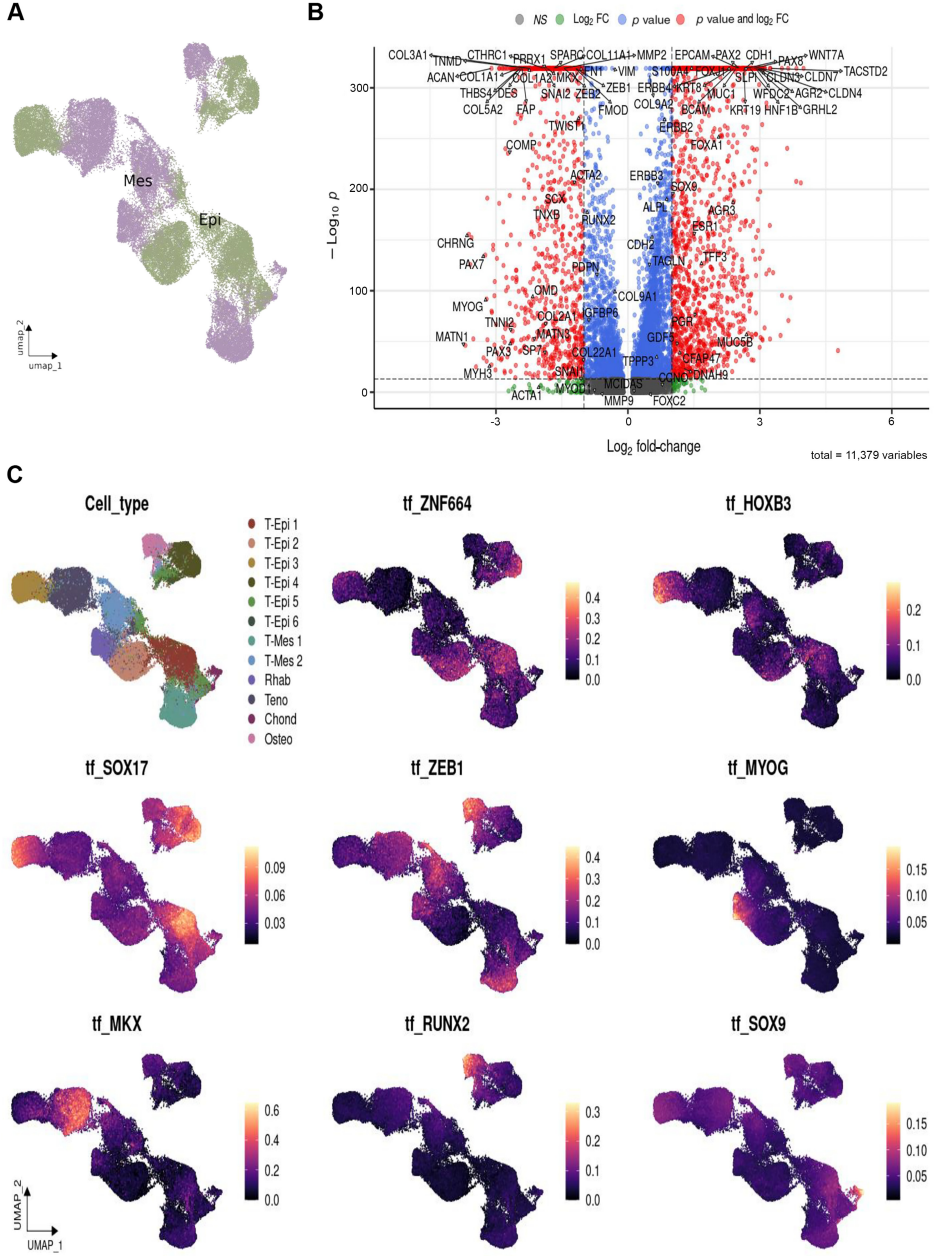
Transcript differences between epithelial and mesenchymal compartments in carcinosarcomas. (A) UMAP plot highlighting distinct clustering of epithelial (green) and mesenchymal (lilac) populations. (B) Volcano plot showing differentially expressed genes between epithelial and mesenchymal cell populations across six carcinosarcoma (CS) samples. Genes highlighted in red meet both fold‐change and adjusted *p* value thresholds. (C) UMAP visualizations showing TF activity projected onto the integrated single‐nucleus landscape. These patterns provide functional evidence for the co‐existence of multiple differentiation trajectories within the mesenchymal compartment of CSs.

To explore the molecular differences between the epithelial and mesenchymal compartments of CSs (Figure 2A), we performed differential gene expression analysis across six cases. This revealed divergent transcriptomic profiles for each cellular phenotype (Figure 2B and supplementary material, Table S5). The epithelial component exhibited overexpression of canonical epithelial genes such as *PAX8*, *CDH1*, *EPCAM*, *BCAM*, *WNT7A*, *WFDC2*, *S100A6*, and *MUC1*, as well as various keratins (*KRT8*, *KRT19*) and claudins (*CLDN3*, *CLDN4*, *CLDN7*), all associated with epithelial identity, adhesion, and tumor progression.

The mesenchymal compartment showed marked upregulation of extracellular matrix and stromal remodeling genes, including *COL3A1*, *COL5A2*, *COL11A1*, *SPARC*, *FAP*, *CTHRC1*, and *MMP2*. Core transcriptional regulators of mesenchymal identity and EMT, such as *PRRX1*, *ZEB1*, and *TWIST1*, were also significantly overexpressed. Several lineage‐specific programs emerged within the mesenchymal population: *MYOG* was indicative of myogenic differentiation; *RUNX2* and *SP7* pointed toward osteogenic traits; *SOX9* indicated chondrogenic differentiation; and *TNMD* was consistent with a tenogenic signature. These patterns collectively highlight the molecular heterogeneity and plasticity of the mesenchymal compartment in CSs.

We performed a comparative analysis of regulatory transcription factor (TF) activity between epithelial and mesenchymal populations (Figure 2C). The epithelial compartment was enriched in TFs known to participate in epithelial developmental and maintenance programs (*ZNF444*, *ZNF664*, *HOXB3/6*, *HOXA1/4*, *FOXL1*, *MESP1*, *SOX17*, *ID4*, *GRHL2*, *EHF*, *PAX8*, and *PAX2*). In the mesenchymal compartment, core EMT regulators such as *ZEB1*, *TWIST1*, *TWIST2*, and *TCF4* were prominently upregulated, consistent with a transition away from epithelial identity. Notably, lineage‐specific regulators were also enriched (Figure 2C).

### Neoplastic cells with epithelial (carcinomatous) differentiation

Six epithelial tumor populations (T‐Epi 1–6) were identified across the cases, each showing distinct molecular profiles as well as differentially upregulated biological processes, as revealed by gene ontology (GO) enrichment analysis (supplementary material, Figures S2 and S3). Clustering by sample of origin confirmed that some epithelial populations were case‐specific, while others, such as T‐Epi 1 and T‐Epi 5, were shared across tumors, although with varying abundance (supplementary material, Figure S4).

These neoplastic epithelial clusters exhibited heterogeneous transcriptional profiles but consistently retained partial expression of canonical epithelial markers (supplementary material, Table S4). Core genes such as *CDH1*, *EPCAM*, and *BCAM*, along with tight junction components including *CLDN3*, *CLDN4*, *CLDN7*, and *TACSTD2*, were variably expressed across the clusters, reflecting a gradient of epithelial identity retention. Additionally, structural epithelial genes such as *KRT7* and *KRT8* were broadly present, supporting the epithelial origin of these neoplastic populations. The expression of *WFDC2*, a secretory marker frequently upregulated in gynecological carcinomas, as well as transcription factors *PAX2* and *PAX8*, further reinforces the association of these populations with Müllerian epithelial lineages, albeit with evidence of partial loss, dedifferentiation, or lineage deviation in specific clusters.

Further characterization of all epithelial subpopulations, including defining markers, case‐specific distributions, and functional annotations, is provided in the Supplementary materials and methods.

### Neoplastic cells with mesenchymal (sarcomatous) differentiation

All CSs analyzed exhibited a prominent mesenchymal compartment, consistent with their biphasic nature. Transcriptomic clustering revealed the presence of two general mesenchymal populations, T‐Mes 1 and T‐Mes 2, alongside lineage‐specific populations associated with rhabdomyogenic (Rhab), tenogenic (Teno), osteogenic (Osteo), and chondrogenic (Chond) differentiation. These mesenchymal clusters showed distinct molecular identities, upregulation of specific biological processes, and a heterogeneous distribution across tumors (supplementary material, Figures S5 and S6, and Table S4).

Among the mesenchymal clusters identified, T‐Mes 2 emerged as the most conserved population, being present across all six CSs, with a higher proportion observed in the three cases with rhabdomyogenic differentiation (CS1–CS3). This cluster displayed a robust mesenchymal transcriptional profile, defined by high expression of structural and matrix‐related genes such as *COL1A1*, *SPARC*, and *CTHRC1*. These genes are hallmark indicators of fibroblast‐like identity and extracellular matrix remodeling, consistent with a tumor‐associated mesenchymal phenotype. Additionally, *CDH11*, involved in mesenchymal adhesion and motility, was expressed, reinforcing the migratory and invasive potential of this population. Interestingly, T‐Mes 2 also showed strong expression of *SNAI2* (Slug), a master regulator of EMT, along with *FN1* (fibronectin), a major extracellular matrix component typically upregulated during EMT processes. This suggests that T‐Mes 2 not only embodies a fibroblast‐like identity but may also reflect an active EMT program contributing to the phenotypic plasticity and invasiveness of CSs.

In contrast, T‐Mes 1 was exclusively found in the chondrogenic CS of ovarian origin (CS6), highlighting its tumor‐specific nature. Although it shared the expression of general mesenchymal genes such as *COL1A2* and *CDH11*, T‐Mes 1 displayed a distinct transcriptional profile, suggestive of a more differentiated or tissue‐restricted state. Notably, T‐Mes 1 showed high expression of *DLK1*, *FDX1*, and *APOD*, genes implicated in mesenchymal and ovarian stromal function, including lipid metabolism and progenitor cell regulation, supporting the notion that this population reflects a tumor‐specific mesenchymal program influenced by the ovarian microenvironment. Although CS6 also contained the broadly distributed T‐Mes 2 population, the presence of T‐Mes 1 underscores the capacity of CSs to establish distinct stromal niches reflective of their tissue of origin.

### Sarcomatous lineage specialization across carcinosarcomas

Regarding the specific mesenchymal differentiations observed across the CS cohort, most cases (CS1–CS3) exhibited rhabdomyogenic differentiation. Interestingly, although CS2 had initially been diagnosed as homologous CS, snRNA‐seq revealed a small but distinct population of rhabdomyoblasts (Rhab), which was subsequently confirmed by MyoD1 and myogenin IHC.

**Figure 3:**
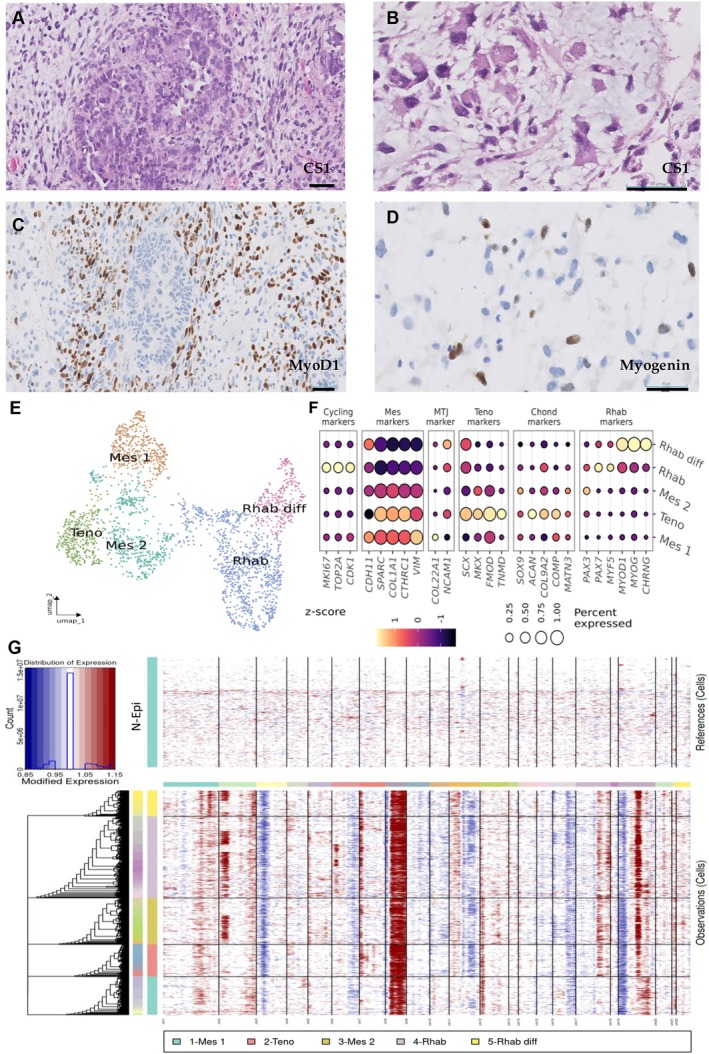
Characterization of CS1. (A, B) H&E staining showing biphasic morphology in CS1, with an epithelial component and a mesenchymal rhabdoid component (scale bar, 50 μm). (C, D) IHC showing expression of MyoD1 and myogenin (scale bar, 50 μm). (E) UMAP plot of mesenchymal populations. (F) Dot plot representing the percentage of expressing cells and average gene expression of canonical markers across mesenchymal clusters. (G) Heatmap showing expression‐based inference of the CNV landscape across malignant mesenchymal cells.

**Figure 4:**
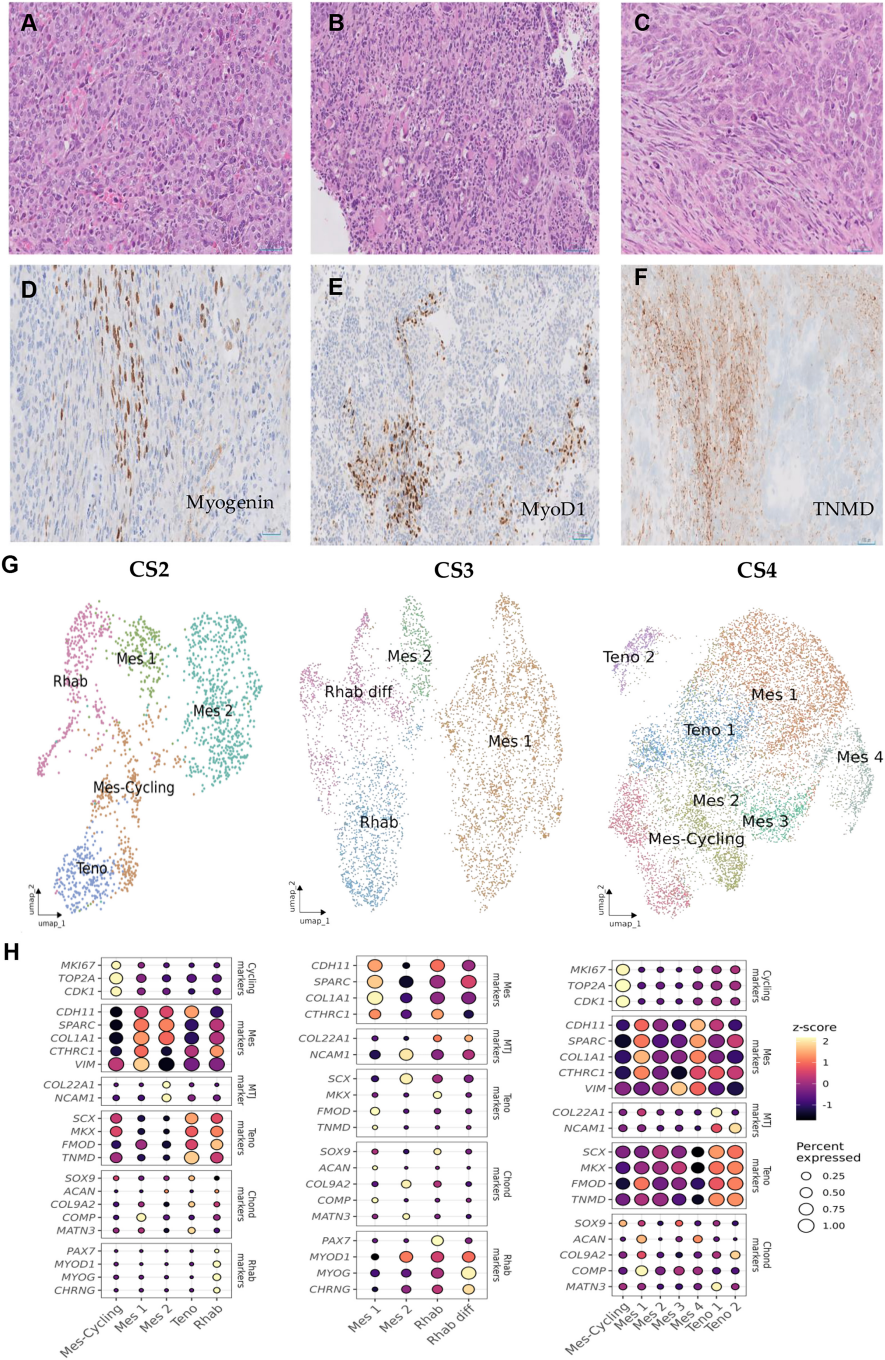
Pathological and mesenchymal analysis of CS2–CS4. (A–C) H&E staining showing biphasic morphology in CS2 (A), CS3 (B), and CS4 (C). (D, E) IHC showing expression of myogenin in CS2 (D) and MyoD1 in CS3 (E). Scale bar, 50–100 μm. (F) ISH of TNMD in CS4. Scale bar, 100 μm. (G) UMAP plots showing the clustering of mesenchymal cell populations. (H) Corresponding dot plots displaying the expression levels (*z*‐score) and percentage of expressing cells for canonical mesenchymal and lineage‐specific markers across clusters, highlighting molecular heterogeneity and differentiation trajectories.

The Rhab population was detected exclusively in CS1–CS3. This cluster exhibited a transcriptional program highly consistent with skeletal muscle lineage commitment. Key myogenic regulators such as *MYOG*, *MYOD1*, and *RBM24* were among the top markers (supplementary material, Table S4), consistent with an active program of myogenic differentiation (Figures 3A–D and 4A,B,D,E). *MYOG* and *MYOD1*, in particular, are master transcription factors driving terminal skeletal muscle maturation. Furthermore, the Rhab cluster expressed *CHRNG*, the fetal‐specific gamma subunit of the nicotinic acetylcholine receptor, which is typically active during early muscle development and neuromuscular junction (NMJ) formation. In addition, several other NMJ‐associated genes were detected in this population, including *CHRNA1*, *CHRNB1*, *CHRND*, *MUSK*, and *RAPSN*, suggesting the activation of a transcriptional program supporting not only rhabdomyogenic identity but also the molecular architecture required for NMJ assembly.

Moreover, sub‐analysis of the mesenchymal component at the individual tumor level revealed rhabdomyoblastic populations with varying degrees of differentiation in CS1 and CS3, suggesting heterogeneity within the skeletal muscle program across tumors. Figures 3E–G and 4G,H illustrate subclustering of the mesenchymal compartment in CSs with rhabdomyoblastic differentiation, where two distinct rhabdomyoblast populations were evident in CS1 and CS3 (Figures 3E and 4G). One cluster displayed a less differentiated profile (Rhab), characterized by the expression of progenitor markers such as *PAX7* and *MYF5*, while the other showed a more mature phenotype (Rhab diff) marked by high expression of *MYOG* (Figures 3F and 4H). In CS1, both populations appeared transcriptionally more similar to the Mes 2 cluster, as supported by their shared CNV patterns shown in Figure 3G, further indicating a common mesenchymal origin.

CS4, which had also initially been diagnosed as a homologous CS, was later found to be heterologous upon detailed analysis, exhibiting a tenogenic differentiation pattern that has not been previously described (Figure 4G,H). snRNA‐seq revealed the presence of a distinct mesenchymal population (Teno) predominantly derived from this tumor. Interestingly, Teno cells were also detected at lower proportions in CSs with rhabdomyogenic differentiation, being relatively more abundant in CS2, the case with the lowest number of Rhab cells, in which individual sub‐analysis revealed a more predominant mesenchymal population (Mes 2) expressing genes associated with the muscle–tendon junction (MTJ), such as *COL22A1* and *NCAM1* (Figure 4G,H).

The Teno population displayed a transcriptional signature highly consistent with tendon lineage differentiation. Key markers of tenogenesis such as *SCX* (scleraxis), *MKX* (Mohawk homeobox), *TNMD* (tenomodulin), and *THBS4* (thrombospondin 4) were among the most highly expressed genes within this cluster (supplementary material, Table S4). *SCX* and *MKX* are master transcription factors essential for the specification and maintenance of tendon progenitor cells, while *TNMD* is a hallmark marker of mature tenocytes, involved in regulating collagen fibril organization. TNMD protein expression was further validated by ISH across CS1–CS4 (Figure 4C,F). The high expression of *THBS4*, associated with extracellular matrix assembly in tendinous tissues, further supported the tenogenic identity of this cluster.

In addition, in the sub‐analysis of CS3 and CS4, a mesenchymal population exhibited co‐expression of markers associated with both chondrogenic (*ACAN* and *COMP*) and tenogenic differentiation (*SCX*, *TNMD*, and *COL1A1*) (Figure 4G,H). These findings may suggest a transitional phenotype related to differentiation toward the tendon–bone junction (enthesis).

The discovery of a tenogenic differentiation program highlights the remarkable lineage plasticity of CSs and expands the known spectrum of heterologous mesenchymal differentiations within these tumors.

To validate these findings, we performed RT‐qPCR for selected tenogenic and interface markers (*TNMD*, *MKX*, *ACAN*, *COL22A1*, *NCAM1*) in cohort cases with available material (CS3, CS4, CS6), in an additional set of CSs (supplementary material, Table S6), and in tendon tissue as a positive control. For CS6, in addition to the block analyzed by snRNA‐seq (CS6A), we also included a separate block (CS6B) enriched in spindle‐shaped mesenchymal areas, reflecting the marked heterogeneity of this tumor. Using normal endometrium as a reference control, we confirmed the expression of *TNMD* and *MKX* in several tumors, including rhabdomyosarcomatous, tenogenic, and chondroid subtypes, indicating that these programs are not restricted to a single histological category. Interface‐associated markers were also detected but were overall heterogeneous across CS subtypes. In contrast, none of these markers were significantly expressed in normal endometrium or in CS10, a lymph node metastasis composed exclusively of epithelial tumor cells, supporting their specificity for mesenchymal tumor components (supplementary material, Figure S7).

The Osteo population in CS5 exhibited a transcriptional profile characteristic of osteoblast lineage commitment. Key markers of osteogenesis, including *RUNX2*, *SP7*, and *OMD*, were among the upregulated genes within this cluster (Figure 1I). *RUNX2* is a master transcription factor essential for early osteoblast differentiation, while *SP7* acts downstream to promote maturation of osteoblasts and bone matrix deposition. The strong expression of *OMD*, a small leucine‐rich proteoglycan involved in collagen fibrillogenesis and mineralization, further reinforced the osteogenic identity of this population. In addition, the presence of osteoclasts and the expression of SATB2 confirm the osteogenic nature of CS5 (supplementary material, Figure S8A,B).

Similarly, a distinct chondroblast population (Chond) was identified in CS6, the ovarian CS (supplementary material, Figure S8C,D). This cluster showed a transcriptional signature consistent with chondrocyte lineage differentiation. High expression of *SOX9*, *ACAN*, *COL9A2*, and *COMP* (Figure 1I and supplementary material, Table S4) defined this cluster. *SOX9* is a master regulator of chondrogenesis, driving the expression of extracellular matrix components critical for cartilage formation. *ACAN* encodes aggrecan, a major proteoglycan in cartilaginous tissue, while *COL9A2* and *COMP* are key structural components of the cartilage extracellular matrix.

### Copy number variations

**Figure 5:**
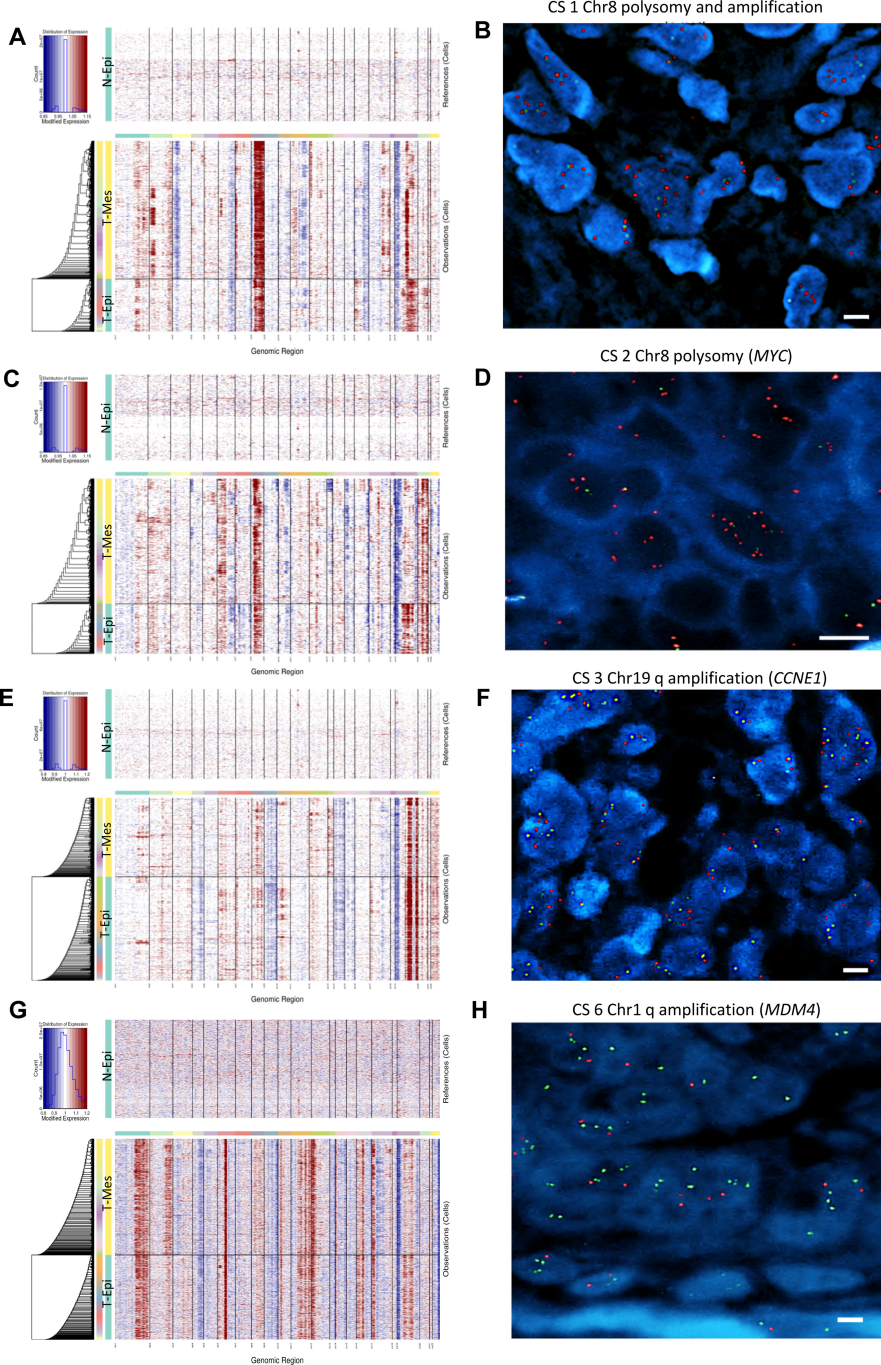
Analysis of copy number variations. (A, C, E, G) Expression‐based inference of copy number variation (CNV) profiles from single‐nucleus RNA‐seq data in CS1 (A), CS2 (C), CS3 (E), and CS6 (G). Tumor‐derived mesenchymal and epithelial cells (T‐Mes, T‐Epi) are compared with normal epithelial references (N‐Epi). Chromosomal gains and losses are shown in red and blue, respectively, across the genome (*x*‐axis). (B, D, F, H) FISH validation of representative CNVs in the same cases. Scale bar, 10 μm. (B) *MYC* (green signals) amplification and chromosome 8 polysomy in CS1. (D) Chromosome 8 (red signals) polysomy in CS2 (*MYC* probe). (F) *CCNE1* amplification (red signals) on chromosome 19q in CS3. (H) *MDM4* (green signals) amplification on chromosome 1q in CS6.

CNVs were detected across all cases in both epithelial and mesenchymal compartments, using proliferative‐phase normal endometrial epithelial cells as the reference (Figure 5 and supplementary material, Figure S9).

The most recurrent event was chromosome 8q amplification, present in all CS samples. While this alteration was homogeneous in some tumors, in others, such as CS4, it was restricted to the mesenchymal component (supplementary material, Figure S9A). Notably, in CS2, CNV analysis revealed amplification of the central region of chromosome 8, and FISH analysis showed centromeric polysomy without amplification of the *MYC* locus itself, as evidenced by the absence of a *MYC*‐specific probe signal (Figure 5D).

Another frequent alteration was chromosome 19q amplification, observed in CS1–CS4 and CS6, with variable compartmental distribution. For example, it was restricted to the mesenchymal fraction in CS1, to the epithelial cells in CS6, and was present in both compartments in CS3. In contrast, loss of chromosome 19p occurred in CS1–CS3 and CS5–CS6. Chromosome 1q amplification was seen in CS6 as well as in mesenchymal subsets of CS1 and CS3.

These CNV findings were partially validated by FISH using probes for *MDM4* (1q), *MYC* (8q), and *CCNE1* (19q), with overall high concordance between platforms (supplementary material, Table S7). Single‐nucleus CNV analysis, however, offered finer resolution, uncovering cell type‐specific alterations and intratumoral heterogeneity that occasionally escaped detection by FISH. For example, *MYC* amplification (8q) in CS4 was detected only in mesenchymal cells by single‐nucleus analysis, but not by FISH.

## Discussion

In this exploratory study, we uncover extensive cellular and molecular heterogeneity across six CSs and normal endometrial controls. Our analysis highlights a diverse landscape of lineage‐specific mesenchymal differentiation programs. Notably, one of the most significant findings of this study was the identification of a previously unrecognized tenogenic differentiation program in three cases, two of them initially diagnosed as homologous CS.

This tenogenic cluster, characterized by the expression of tendon markers (*SCX*, *MKX*, *TNMD*, *THBS4*) [26, 27], was transcriptionally distinct and further validated by TNMD ISH. Interestingly, the presence of tenogenic subpopulations was not limited to the case with exclusive tenogenic differentiation (CS4) but was also observed at lower levels in two rhabdomyogenic CSs, suggesting a shared progenitor or overlapping differentiation pathways. The co‐expression of *COL22A1* and *NCAM1*, markers associated with the MTJ in CS2, further supports the existence of transitional states between closely related mesodermal lineages [27]. Similarly, in CS3 and CS4, the co‐expression of tenogenic (*SCX*, *FMOD*) and chondrogenic markers (*ACAN*, *COMP*, *COL9A1*) [28], as well as enthesis‐specific ECM genes (*COL2A1*), raises the possibility of enthesis‐like differentiation [29], a tendon–bone interface not previously described in CSs.

Building upon this observation, we found that rhabdomyogenic populations also showed expression of key NMJ‐related genes, including *CHRNG*, *CHRNA1*, *CHRNB1*, *MUSK*, and *RAPSN* [30]. This constellation of markers, typically involved in the formation and maintenance of functional NMJs, suggests that some CSs may partially recapitulate developmental programs that govern neuromuscular connectivity. Together with the evidence of MTJ‐like and enthesis‐like transcriptional signatures, these findings expand the known repertoire of mesodermal specialization in CSs and argue against random lineage switching. Instead, they support the presence of structured, interface‐specific differentiation programs, potentially driven by intrinsic progenitor plasticity or microenvironmental patterning cues. These insights warrant further investigation into the signaling pathways and niche factors orchestrating this degree of mesenchymal complexity.

Importantly, our data also revealed intra‐lineage heterogeneity, reflecting different stages of mesenchymal maturation. In CS4, two transcriptionally distinct tenogenic subpopulations (Teno 1 and Teno 2) were identified, possibly representing progenitor and more differentiated tenocyte states. Similarly, in rhabdomyogenic CSs, we observed two subclusters: an early‐stage group expressing *PAX7* and *MYF5*, and a more mature group marked by *MYOG*, consistent with terminal skeletal muscle differentiation. These findings suggest that CSs not only activate lineage‐specific programs but may also preserve hierarchical differentiation trajectories reminiscent of normal developmental or regenerative processes.

This degree of plasticity was not limited to the mesenchymal compartment. Neoplastic epithelial cells showed considerable transcriptional diversity, with six tumor‐specific epithelial clusters variably expressing markers of epithelial identity, secretory function, proliferation, and dedifferentiation. Some clusters displayed hybrid phenotypes, co‐expressing epithelial and mesenchymal markers, consistent with intermediate EMT states. This pattern aligns with the continuum model of EMT, widely described in other carcinomas [31, 32].

Single‐nucleus CNV analysis revealed widespread genomic alterations across both epithelial and mesenchymal compartments, with recurrent gains and losses affecting key chromosomal regions such as 8q, 19q, and 1q. In several cases, these alterations were compartment‐specific, highlighting intratumoral heterogeneity not captured by bulk profiling. These patterns suggest clonal divergence and selective pressures operating within distinct cellular compartments. FISH analyses confirmed several of these findings, reinforcing the structural validity of the inferred CNVs. However, the single‐nucleus approach revealed additional cell type‐specific alterations, such as mesenchymal‐restricted *MYC* amplification in CS4, that were not detected by FISH, likely due to sampling limitations or subclonal representation. These data support a model in which genomic instability fuels transcriptional diversification, enabling CSs to give rise to multiple mesenchymal lineages from a shared neoplastic progenitor.

While our study provides novel insights, it also has several limitations. The analysis was conducted on a small number of cases. The main heterologous components were represented, but other subtypes such as liposarcomatous or angiosarcomatous were not included. Moreover, the data capture only a single time point and therefore do not reflect the dynamic nature of lineage transitions. An additional limitation is that tissue sampling was limited to a single section per case. Although regions with the highest morphological diversity were carefully selected, this approach inevitably represents only a fraction of the tumor's full complexity and intratumoral heterogeneity.

Furthermore, all snRNA‐seq analyses were performed on FFPE samples. While traditionally associated with concerns regarding RNA integrity and cell‐type resolution, recent advances, including our own comparative analysis of FFPE and fixed fresh samples [13], demonstrate that FFPE‐based snRNA‐seq can generate high‐quality data comparable to those obtained from fresh tissue, with sufficient sensitivity to detect even rare and underrepresented populations, such as osteoclast‐like cells in CS5 (supplementary material, Figure S10). Future studies incorporating longitudinal sampling and multi‐omic approaches could further elucidate the complexity of CS differentiation.

**Figure 6:**
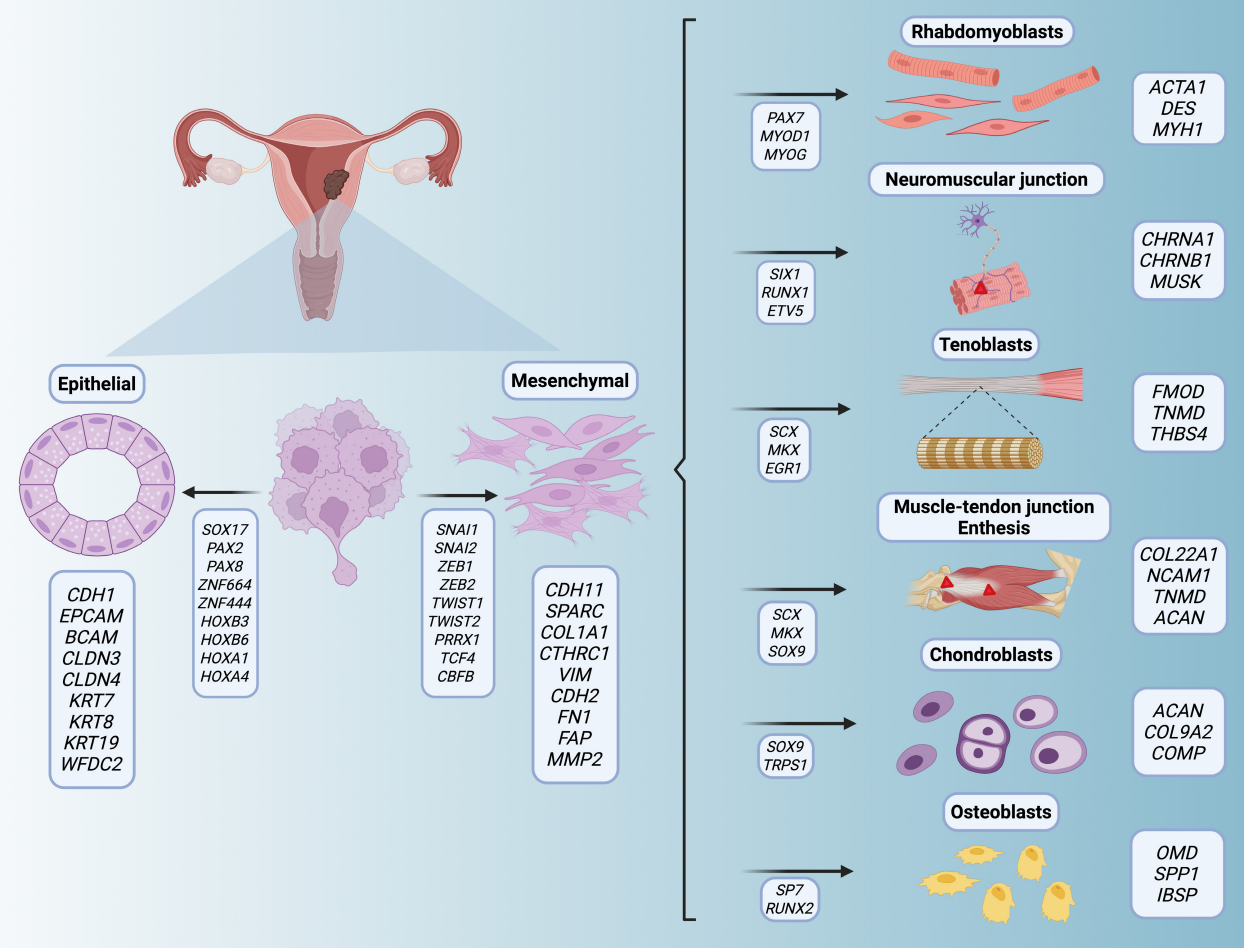
Diagram illustrating the differentiation of epithelial and mesenchymal components in CSs (created in BioRender.com; ID: MD28WHWXEN).

Altogether, our findings expand the current understanding of CS biology by revealing the extent of mesenchymal lineage diversity and the developmental plasticity that underlies it, as is represented in Figure 6. The identification of a previously unrecognized tenogenic program, along with the refined characterization of known heterologous lineages, highlights the potential of single‐nucleus transcriptomics to resolve complex differentiation hierarchies in biphasic tumors. These results suggest that CSs are not defined by fixed epithelial or mesenchymal identities, but rather by dynamic and co‐existing trajectories of lineage specification.

From a diagnostic point of view, our study suggests that the frequency of heterologous CSs tends to be underestimated if biomarker analysis is not performed, mainly if the tumor contains a minor population of heterologous cells. Whereas the presence of relatively differentiated rhadomyoblastic cells can be suspected by morphology, the identification of more immature cells with this type of differentiation can be missed if specific IHC studies (MyoD1, PAX7) are not performed, as in our CS2. Additionally, since the diagnosis of osteosarcoma and chondrosarcoma differentiation relies on the identification of malignant osteoid and cartilage respectively, cases with no or little matrix production, such as our CS5 case, can be missed. Finally, tenogenic differentiation occurs in cells with an unspecific fibroblastic morphology, which have been probably considered as homologous CS until now, such as our CS4 case. Future studies will be needed to determine the utility of performing biomarker analysis in CSs, in order to refine classification and prognosis and the possible prognostic significance of tenogenic differentiation and its contribution to disease progression and treatment response in this aggressive type of tumors.

## Author contributions statement

SG‐M performed the tissue‐based work, bioinformatic analysis, statistical analysis, data interpretation and manuscript writing. JP conceived and designed the study and contributed to pathology review of tumor sections, data interpretation and manuscript writing. IC‐B captured the images of the IHC and H&E‐stained preparations and reviewed the manuscript. VF‐L provided bioinformatic support. AC‐S collected informed consents from patients. JR, XM‐G and SG reviewed the manuscript. JC performed a critical review of the manuscript. BP‐M contributed to the conception and design the study, to case retrieval and IHC, FISH and ISH evaluation. All authors have read and agreed to the final version of the manuscript.

## Supporting information


**Supplementary materials** **and methods**
Supplementary results

**Figure S1.** Subclustering analysis of highly proliferative (cycling) cells across carcinosarcomas (provided in a separate Word document)
**Figure S2**. Bar plots of enriched Biological Process GO terms among upregulated genes in epithelial populations (provided in a separate Word document)
**Figure S3**. Gene ontology network (cnet) plots of enriched Biological Process terms among upregulated genes in epithelial populations (provided in a separate Word document)
**Figure S4**. UMAP visualization of epithelial clusters split by sample of origin (provided in a separate Word document)
**Figure S5**. Bar plots of enriched Biological Process GO terms among upregulated genes in mesenchymal populations (provided in a separate Word document)
**Figure S6**. Gene ontology network (cnet) plots of enriched Biological Process terms among upregulated genes in mesenchymal populations (provided in a separate Word document)
**Figure S7**. Relative expression (log_2_ fold‐change) of tenogenic (*TNMD*, *MKX*) and interface markers (*ACAN*, *COL22A1*, *NCAM1*) by RT‐qPCR (provided in a separate Word document)
**Figure S8**. Histological and immunophenotypic characterization of CS5 and CS6 (provided in a separate Word document)
**Figure S9**. Inferred copy number variation profiles of mesenchymal and epithelial cells in CS4 and CS5 (provided in a separate Word document)
**Figure S10**. UMAP plots of individual carcinosarcoma samples (CS1–CS6) showing tumor and microenvironment composition (provided in a separate Word document)

**Table S1.** Antibodies used for immunohistochemistry (provided as a separate Excel file)
**Table S2**. Single‐nucleus sequencing cell calling and mapping quality metrics (provided as a separate Excel file)
**Table S3**. Cell type distribution across carcinosarcoma cases and normal samples (provided as a separate Excel file)
**Table S4**. Top cluster marker genes identified by differential expression analysis (provided as a separate Excel file)
**Table S5**. Top mesenchymal and epithelial marker genes identified by differential expression analysis (provided as a separate Excel file)
**Table S6**. Clinicopathological characteristics of cases included for validation (provided as a separate Excel file)
**Table S7**. Concordance between scRNA‐seq and FISH for gene amplifications in *MDM4* (1q), *MYC* (8q), and *CCNE1* (19q) across CS1–CS6. Darker colors indicate discordances (provided as a separate Excel file)

## Data Availability

The snRNA‐seq data from this study are available through the Gene Expression Omnibus under accession number GSE299623. Code related to the analyses in this study can be found on GitHub at https://github.com/Gonzalez-Martinez/Carcinosarcomas-study.git.
