# Supplementary material for: Single‐nucleus RNA sequencing identifies a novel tenogenic heterologous differentiation in endometrial carcinosarcomas: implications for diagnosis and tumor classification
Source: J Pathol. 2026 Jan 15;268(2):227–42. doi: 10.1002/path.70003 (PMC12805614; doi:10.1002/path.70003)
Supplement: Supplementary file 1 — Supplementary materials and methods Supplementary results [file PATH-268-227-s003.docx]

**Single-nucleus RNA sequencing identifies a novel tenogenic heterologous differentiation in endometrial carcinosarcomas: implications for diagnosis and tumor classification**

S González-Martínez *et al. J Pathol* [https://doi.org/10.1002/path**.**70003](https://doi.org/10.1002/path.70003)

**Supplementary materials and methods**

**Supplementary results**

**Supplementary Figures S1–S10 are provided in a separate Word document**

**Supplementary Tables S1–S7 are provided as a separate Excel file**

Reference numbers refer to the main text list

**Supplementary materials and methods**

**Pathological and molecular characterization of carcinosarcomas**

Immunohistochemistry (IHC) was carried out on a Leica BOND-MAX autostainer using the BOND Polymer Refine Detection Kit (Leica Biosystems, Richmond, IL, USA). Antibodies included p53, myogenin, MyoD1, CD8, CD68, STAB2, S100A, PAX8, and desmin. Clones and suppliers are presented in supplementary material, Table S1. RNA ISH was carried out using RNAscope™ 2.5 LS Probe – Hs-TNMD (#564408).

FISH was performed using ZytoVision probes for *MDM4*, *CCNE1*, and *MYC* Dual Color Probe Kit (ZytoVision GmbH, Bremen, Germany). FISH slides were observed using a fluorescence microscope at 100× with immersion oil. The whole slide was reviewed to assess heterogeneity and a detailed scoring of at least 20 neoplastic cells per sample was performed. Amplification was considered when the tumor cell population had at least twice as many gene signals as centromere signals of the respective chromosome (ratio ≥ 2).

For massive parallel sequencing, ten sections of 10 μm each were cut per case from the same blocks from which material was obtained for scRNA-seq analysis. Sequencing of DNA was carried out as previously reported [22].

**Processing of FFPE tissue samples for scRNA-seq**

FFPE tissue samples were processed following the protocol ‘Isolation of Cells from FFPE Tissue Sections for Chromium Fixed RNA Profiling’ (CG000632 from 10x Genomics, Pleasanton, CA, USA). Tissue dissociation was carried out using the gentleMACS Octo Dissociator with Heaters (Miltenyi Biotec, Auburn, CA, USA), utilizing the pre-installed program 37C_FFPE_1 for approximately 48 min as specified in the protocol. Once the dissociation protocol was completed, the cells were counted and frozen using the same procedure as that for cells derived from fresh samples. In this case, the cells were kept at −80 ºC for only 1 week before commencing the ‘Chromium Fixed RNA Profiling Reagent Kits for Singleplexed Samples protocol’ (CG000477 from 10x Genomics).

**10x library preparation and sequencing**

The samples were thawed and recounted. Single-cell library preparation was conducted following the manufacturer’s protocol for the Chromium Fixed RNA Profiling Reagent Kits for Singleplexed Samples (CG000477 from 10x Genomics). The cells were loaded onto the Chromium X/iX to generate single-cell gel beads in emulsions (GEMs). Subsequently, the libraries were sequenced on a NovaSeq 6000 system (Illumina, San Diego, CA, USA) with approximately 200 million reads per library.

**scRNA-seq data processing**

Sequencing reads were aligned against the reference transcriptome GRCh38, and unique molecule identifiers (UMIs) were quantified using Cell Ranger, version 7.1.0 (10x Genomics). Subsequent analyses were performed using R (version 4.3.2) and the Seurat package (version 5.0.2). Gene expression data of all samples were merged and filtered for the following quality parameters: cells with more than 200 detected genes, a minimum log_10_(genes per UMI) ratio of 0.8, and a mitochondrial read fraction below 20%. Subsequently, the doublets were analyzed and removed using doubletFinder (version 2.0.4) [23], and a total of 96,298 cells remained (supplementary material, Table S3).

Gene expression data were normalized using the SCTransform() function, regressing out mitochondrial gene expression. Dimensionality reduction was performed using PCA followed by UMAP on the top 30 principal components. Cells were annotated based on canonical marker genes from the literature. Subclustering was performed (resolution = 0.2–0.5) for detailed characterization of epithelial, mesenchymal, and immune populations. Differentially expressed genes were identified using FindAllMarkers (only.pos = TRUE, min.pct = 0.1, logfc.threshold = 0.25).

**Inference of copy number variation (CNV) from single-cell RNA-seq**

In each tumor, putative copy number events were inferred for each epithelial cell cluster using the R package inferCNV version 1.23.0 [33], using epithelial normal cells from healthy donors as baselines to estimate the CNVs of malignant cells. Genes were sorted by their genomic locations on each chromosome. The standard inferCNV algorithm was invoked with infercnv::run() with the cutoff set to ‘0.1’, denoise set to ‘TRUE’, and HMM set to ‘TRUE’. The default i6 Hidden Markov Model (HMM) was used to predict CNV levels based on a six-state CNV model ranging from complete loss to more than two copies. The Bayesian Network Latent Mixture Model was used to estimate the posterior probability of each CNV level at each predicted CNV region.

**Regulon activity inference**

Regulon activity analysis was performed using the pySCENIC pipeline (<https://github.com/aertslab/pySCENIC>). Seurat objects containing epithelial and mesenchymal cells of each case were converted into an expression matrix before being loaded into Python (version 3.6.13). Gene expression data from epithelial and mesenchymal cells were processed to identify transcription factor co-expression modules and their target genes using GRNBoost2. Subsequently, RcisTarget was applied to perform motif analysis and refine the identified regulons. Finally, AUCell was used to assess the activity of these regulons in individual cells, assigning an area under the curve (AUC) score to indicate the activation status of each regulon in each cell. Visualization plots were generated from these results using the SeuratExtend package (version 1.1.3), providing insight into regulon activity across different cell populations.

**Supplementary results**

**Clinicopathological features of the samples**

In addition, molecular data from next-generation sequencing (NGS) in three cases (CS1, CS2, CS6) and IHC in all cases were available. *TP53* alterations were observed in five of the six tumors, either at the genomic level or by IHC. CS1 was the only one that showed a wild-type p53 IHC pattern, suggesting preserved *TP53* function. In contrast, CS2, CS3, CS4, CS5, and CS6 exhibited mutant p53 staining patterns. Additionally, CS2 and CS6 harbored *TP53* mutations identified by NGS (c.786del and p.Cys176Tyr, respectively). Additional NGS findings included activating mutations in the PI3K pathway in CS2 (*PIK3CA* V346G, *PIK3R1* Q432), as well as a mutation in *PPP2R1A* (P179R) (Table 1).

**Cycling cell heterogeneity in carcinosarcomas**

A distinct cluster of highly proliferative cells was identified across the dataset (Cycling cluster), characterized by a transcriptional program consistent with active cell cycling (Figure 1B). The Cycling cluster showed strong overexpression of canonical cell cycle regulators such as *MKI67*, *TOP2A*, *CDK1*, *AURKB*, and *FOXM1*. In addition, genes involved in mitotic spindle assembly (*NUSAP1*, *KIF23*, *PLK1*, *CENPF*) and chromatin organization (*HIST1H1A/B/C*, *HJURP*, *ASF1B*) were also prominently expressed, supporting the mitotic activity of this population (supplementary material, Table S4). This profile indicates a robust proliferative compartment that spans both epithelial and mesenchymal compartments, encompassing cells from multiple tumors (Figure 1D).

To further dissect this proliferative component, we performed a sub-analysis restricted to the Cycling cluster. This revealed seven transcriptionally distinct cycling subpopulations, reflecting both epithelial and mesenchymal differentiation contexts (supplementary material, Figure S6). Among them, two were epithelial: T-Epi-Cycling 1 and T-Epi-Cycling 2, corresponding to CS6 and CS4, respectively. These epithelial cycling clusters retained partial expression of epithelial markers while maintaining strong proliferative signatures.

The remaining five clusters reflected cycling mesenchymal cells: NST-Cycling originated from the stromal compartment of the normal endometrial samples; Teno-Cycling 1 and 2, detected in CS4 and CS2, displayed expression profiles aligned with tendon lineage differentiation; Rhab-Cycling, present in CS1 and CS3, exhibited a myogenic proliferative program; and Osteo-Cycling, identified in CS5, expressed markers characteristic of early dividing osteogenic progenitors. Together, these subpopulations illustrate the lineage-specific diversity within the cycling compartment of the tumor and surrounding stroma (supplementary material, Figure S1).

**Normal proliferative endometrium**

The normal proliferative endometrium was clearly identified in both analyzed samples. In each case, a single homogeneous epithelial population was observed, characterized by the expression of canonical epithelial markers such as *CDH1*, *ESR1*, *SOX17*, or *SPDEF.* Additionally, the epithelial cells expressed genes associated with the proliferative phase of the endometrium, including *KMO*, *IHH*, and *EMID1*, consistent with previously described transcriptional profiles during this stage of the menstrual cycle [25].

In the stromal compartment, two distinct clusters of normal stromal cells (NST 1 and NST 2) were identified in both samples. These clusters exhibited a characteristic gene expression pattern, including stromal and extracellular matrix-related genes such as *FOXL2*, *MMP11*, *CDH11*, *SPARC*, and *COL1A1.*

Moreover, both samples showed the presence of a third stromal population composed of actively cycling cells (Figure S1). This cycling population was more abundant in sample N1, as illustrated in Figure 1D.

**Comparative analysis of transcription factor activity between epithelial and mesenchymal populations**

To further analyze the regulatory mechanisms underlying these differences, we performed a comparative analysis of transcription factor (TF) activity between epithelial and mesenchymal populations (Figure 2C). The epithelial compartment was enriched in several zinc finger proteins (*ZNF444*, *ZNF664*) and homeobox genes (*HOXB3*, *HOXB6*, *HOXA1*, *HOXA4*), known to participate in developmental and epithelial programs. Additional TFs linked to epithelial lineage specification and maintenance included *FOXL1*, *MESP1*, *SOX17*, and *ID4.* The robust expression of *GRHL2*, *EHF*, *PAX8*, and *PAX2*, key regulators of epithelial identity, further reinforced the integrity of the epithelial transcriptional network.

In contrast, the mesenchymal compartment displayed a complex and developmentally enriched transcriptional landscape. Core EMT regulators such as *ZEB1*, *TWIST1*, *TWIST2*, and *TCF4* were prominently upregulated, consistent with a transition away from epithelial identity. The analysis also revealed TFs associated with mesenchymal and stromal function, including *SIX2*, *MAFB*, *FOXF2*, *CBFB*, and *CEBPD*, alongside regulators of vascular or neural crest-related lineages such as *FLI1*, *HIC1*, and *GLIS1*. Additionally, transcription factors such as *MEOX2*, *PLAGL1*, *CREB3L1*, *NR2F2*, and *SP9* highlighted the diversity and plasticity of the mesenchymal regulatory program. Notably, lineage-specific regulators such as *MYLK*, *MKX*, and *SP7* were also enriched, pointing toward rhabdomyogenic, tenogenic, and osteogenic differentiation pathways, respectively.

Finally, to assess the distribution of these transcriptional programs, we visualized the activity of key lineage-specific TFs across the single-cell UMAP embedding (Figure 2D). *ZNF664*, *HOXB3*, and *SOX17* were broadly expressed throughout the epithelial fraction*. ZEB1* was broadly expressed throughout the mesenchymal fraction, consistent with its role in driving EMT. In contrast, TFs associated with terminal differentiation displayed cluster-specific localization: *MYOG* was restricted to the Rhab population (rhabdomyogenic), *RUNX2* was enriched in Osteo (osteogenic), *SOX9* in Chond (chondrogenic), and *MKX* in Teno (tenogenic). These patterns provide functional evidence for the co-existence of multiple differentiation trajectories within the mesenchymal compartment of CSs.

**Neoplastic cells with epithelial (carcinomatous) differentiation**

T-Epi 1 represented the most predominant epithelial population and displayed the strongest epithelial identity among all tumor clusters. It was highly enriched in two CSs with rhabdomyogenic differentiation (CS1 and CS2), as well as in the ovarian CS (CS6). This cluster was characterized by high expression of *MSLN* (mesothelin), a glycoprotein frequently overexpressed in aggressive carcinomas and associated with enhanced invasiveness and poor prognosis. *CLDN6* also marked this population. Additional upregulated genes included *C6orf132* and *ATF3*, both involved in cellular stress responses and cell cycle control; *ATF3* in particular has been implicated in tumor progression and adaptation to oncogenic stress. Notably, *TAGLN* (transgelin), a marker of smooth muscle differentiation often upregulated during epithelial–mesenchymal plasticity, was also expressed, suggesting partial acquisition of mesenchymal traits. Furthermore, *LAMB3*, encoding a laminin β3 subunit involved in basement membrane organization and epithelial cell migration, supports the notion of active remodeling and potential motility within this epithelial compartment.

T-Epi 5, along with T-Epi 1, was one of the only epithelial populations consistently detected across all CSs, although T-Epi 5 was predominant only in CS2. This population displayed a mixed epithelial and secretory profile. Genes such as *CDC20B* and *CALB2*, associated with cell cycle regulation and neuroendocrine-like differentiation, further highlighted the hybrid character of this population. The expression of *PGA5* and *MUC4*, mucin-related genes often found in secretory or poorly differentiated epithelial tumors, supports a partially dedifferentiated phenotype.

The remaining four epithelial populations (T-Epi 2, T-Epi 3, T-Epi 4, and T-Epi 6) exhibited a tumor-specific distribution. Their restricted presence underscores the high degree of intratumoral heterogeneity and highlights the divergent epithelial programs that can emerge within CSs. T-Epi 2 was restricted to CS3. Among its defining markers, *CDH16*, a cadherin typically restricted to renal epithelium and absent in the endometrial lineage, strongly indicated a deviation from normal tissue identity. The co-expression of *DNER*, *CPS1*, and *GRB14* suggested the engagement of developmental and metabolic pathways.

T-Epi 3, identified in the tenogenic CS (CS4), displayed a transcriptional signature indicative of dedifferentiation, metabolic activation, and invasiveness. This population expressed *KRT23* and *MMP7*, both associated with aggressive tumor phenotypes. High levels of *SLC2A1*, a glucose transporter, and *MUC1*, a membrane-bound mucin involved in immune evasion and signaling, supported a metabolically active and secretory phenotype. Additionally, *L1CAM*, a neural adhesion molecule often linked to poor prognosis and metastatic potential, was strongly expressed, along with *WNT7A*, a member of the Wnt family with known roles in proliferation and lineage control. Altogether, this profile suggested a highly plastic and potentially migratory epithelial state.

T-Epi 4 was restricted to the osteogenic CS (CS5) and was defined by genes involved in cytoskeletal remodeling, motility, and immune interaction. *SYBU* and *SEMA3E* are regulators of cytoskeletal organization and cell migration, the latter being known to influence tumor invasiveness through semaphorin signaling. This cluster also expressed *VTCN1* (also known as B7-H4), an immune checkpoint molecule frequently upregulated in epithelial tumors and implicated in immune evasion. Finally, T-Epi 6 represented a very small epithelial population restricted to CS5, with characteristic expression of *KRT5*, *MMP13*, *KLK8*, *C1orf158*, *DMRT1*, *SLC30A2*, *EFHB*, and *IL5RA*.

**Immune cells**

Among the immune cell populations identified across samples, macrophages were the most abundant, with a notably higher prevalence in CSs compared with normal endometrial tissues. Two transcriptionally distinct macrophage subtypes were delineated based on the expression of SPP1 (osteopontin): SPP1⁻ macrophages (SPP1⁻ M) and SPP1⁺ macrophages (SPP1⁺ M).

SPP1⁻ M were the predominant subset and were present across both malignant and non-malignant samples. These cells expressed classical markers such as *CD68*, *CD163*, and *MRC1*, consistent with a tissue-resident, anti-inflammatory, or alternatively activated (M2-like) phenotype. Their presence in both compartments suggests a homeostatic role or stromal-supportive function that remains relatively unaltered by the tumor microenvironment.

In contrast, SPP1⁺ M were only present in tumor samples and were absent from normal tissue. These cells expressed *SPP1* along with other genes such as *GPNMB*, *TREM2*, and *HMOX1*, which are associated with tissue remodeling, immunoregulation, and stress responses. These SPP1⁺ M have been described across multiple tumor types and are characterized by conserved features, including the promotion of fibrosis, ECM remodeling, and immune modulation, traits frequently associated with poor clinical outcomes [34–36]. While they do not show a strongly pro-inflammatory profile, their gene expression suggests that they may support tumor growth by shaping the local environment, helping tumors to evade immune responses and remodel surrounding tissues. Their tumor-specific presence points to a specialized macrophage state that likely contributes to the progression and immune evasion of CSs.

In addition to macrophages, a distinct population of cells with transcriptional features consistent with osteoclasts was identified, specifically enriched in CS5, the CS with osteogenic differentiation (Figure 1J and supplementary material, Figure S6). These cells expressed high levels of key osteoclast-associated genes such as *ACP5* (encoding TRAP), *CTSK* (cathepsin K), *DCSTAMP*, and *ATP6V0D2*, which are involved in bone matrix degradation and cell–cell fusion. The presence of *ITGB3* and *TNFRSF11A* (RANK), both important for osteoclast activation and differentiation, further supports their identity. This highlights a potential functional coupling between mesenchymal differentiation and immune niche specialization, whereby lineage-specific differentiation programs might shape, or be shaped by, immune cell composition. Together, these observations suggest that the immune landscape in CSs is not merely reactive but may participate in, and even reinforce, lineage-specific differentiation and tumor evolution.

Other immune cell types were also detected. These included T lymphocytes, plasma cells, and neutrophils, which were variably distributed across samples. While not the focus of this study, their presence underscores the heterogeneity of the immune landscape in CSs.
