# Supplementary material for: Single‐nucleus RNA sequencing identifies a novel tenogenic heterologous differentiation in endometrial carcinosarcomas: implications for diagnosis and tumor classification
Source: J Pathol. 2026 Jan 15;268(2):227–42. doi: 10.1002/path.70003 (PMC12805614; doi:10.1002/path.70003)
Supplement: Supplementary file 2 — Figure S1. Subclustering analysis of highly proliferative (cycling) cells across carcinosarcomas (provided in a separate Word document) Figure S2. Bar plots of enriched Biological Process GO terms among upregulated genes in epithelial populations (provided in a separate Word document) Figure S3. Gene ontology network (cnet) plots of enriched Biological Process terms among upregulated genes in epithelial populations (provided in a separate Word document) Figure S4. UMAP visualization of epithelial clusters split by sample of origin (provided in a separate Word document) Figure S5. Bar plots of enriched Biological Process GO terms among upregulated genes in mesenchymal populations (provided in a separate Word document) Figure S6. Gene ontology network (cnet) plots of enriched Biological Process terms among upregulated genes in mesenchymal populations (provided in a separate Word document) Figure S7. Relative expression (log2 fold‐change) of tenogenic (TNMD, MKX) and interface markers (ACAN, COL22A1, NCAM1) by RT‐qPCR (provided in a separate Word document) Figure S8. Histological and immunophenotypic characterization of CS5 and CS6 (provided in a separate Word document) Figure S9. Inferred copy number variation profiles of mesenchymal and epithelial cells in CS4 and CS5 (provided in a separate Word document) Figure S10. UMAP plots of individual carcinosarcoma samples (CS1–CS6) showing tumor and microenvironment composition (provided in a separate Word document) [file PATH-268-227-s001.docx]

**Single-nucleus RNA sequencing identifies a novel tenogenic heterologous differentiation in endometrial carcinosarcomas: implications for diagnosis and tumor classification**

S González-Martínez *et al. J Pathol* [https://doi.org/10.1002/path**.**70003](https://doi.org/10.1002/path.70003)

**Supplementary Figures S1–S10**


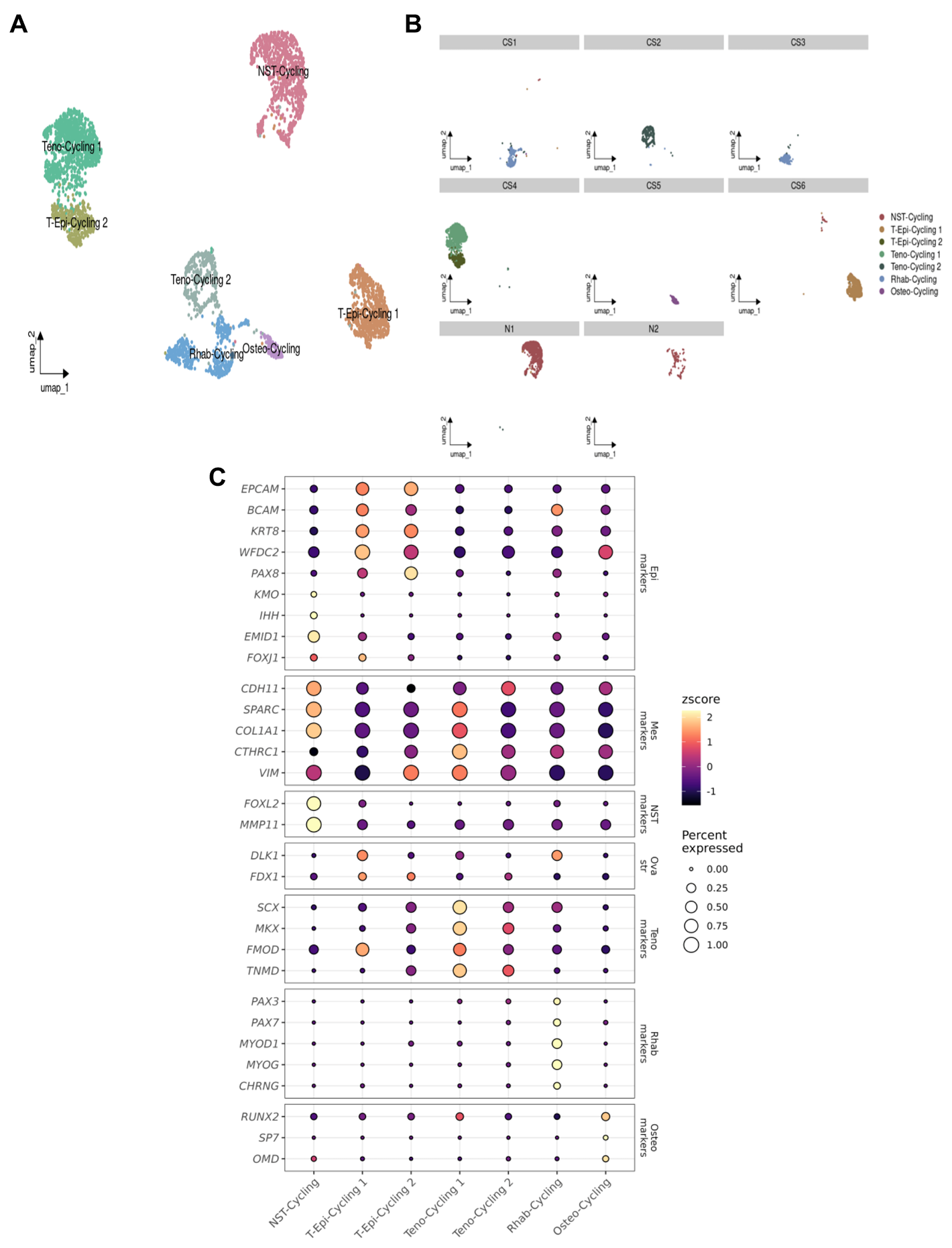


**Figure S1.** **Subclustering analysis of highly proliferative (cycling) cells across carcinosarcomas.** (A) UMAP plot showing seven transcriptionally distinct cycling subclusters identified within the global cycling population: NST-Cycling, T-Epi-Cycling 1–2, Teno-Cycling 1–2, Rhab-Cycling, and Osteo-Cycling. (B) Sample-wise UMAP projections showing the distribution of each cycling subcluster across individual CS tumors (CS1–CS6) and normal endometrial samples (N1, N2). (C) Dot plot representing the average expression and proportion of cells expressing epithelial, mesenchymal, and lineage-specific markers across cycling subpopulations, highlighting functional and phenotypic heterogeneity within the proliferative compartment. NST, normal stromal cells; T-Epi, tumoral epithelial cells; Teno, tenoblasts; Rhab, rhabdomyoblasts; Osteo, osteoblasts.


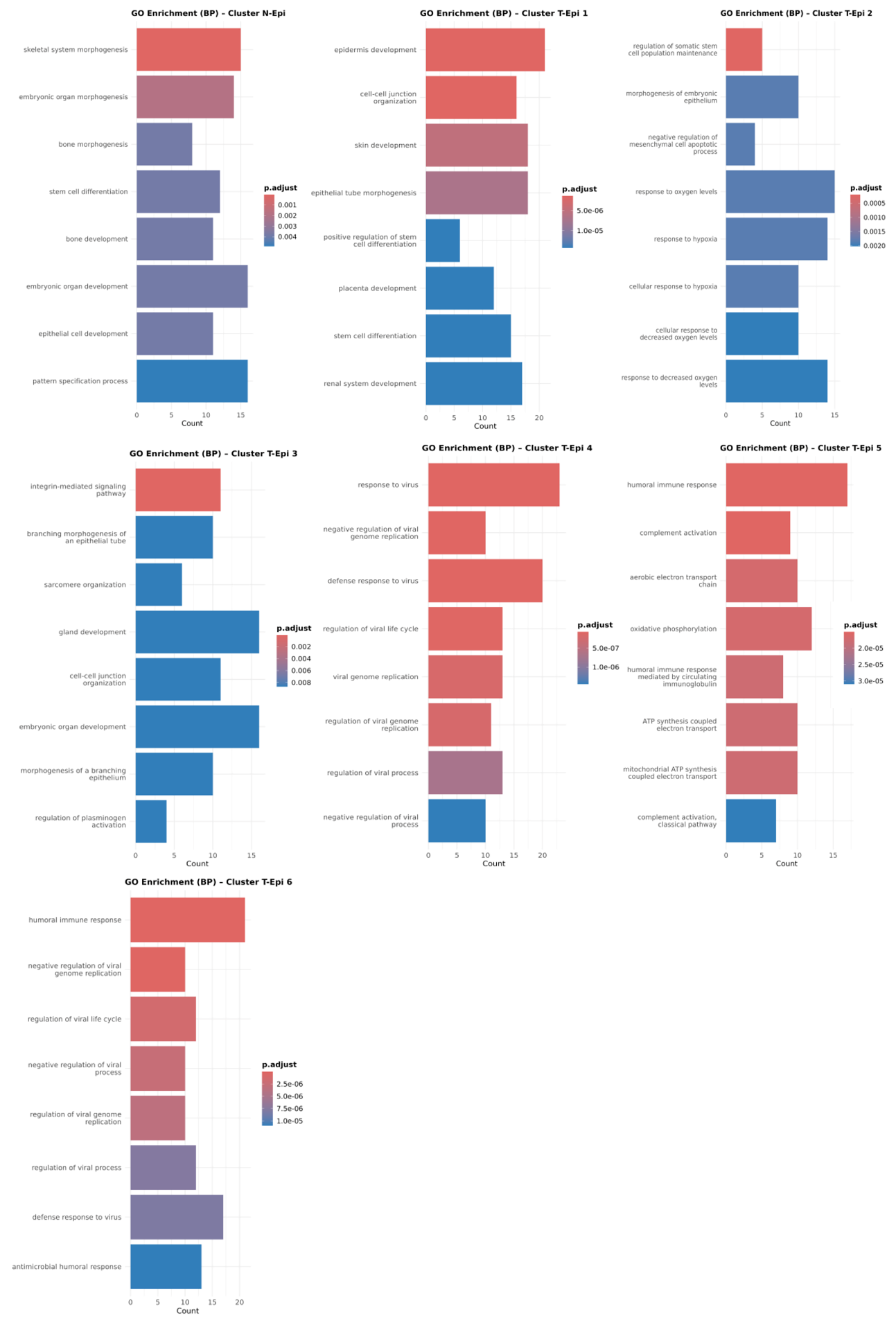


**Figure S2.** **Bar plots of enriched Biological Process GO terms among upregulated genes in epithelial populations.** Color scale represents statistical significance (adjusted *p* value < 0.05).

**
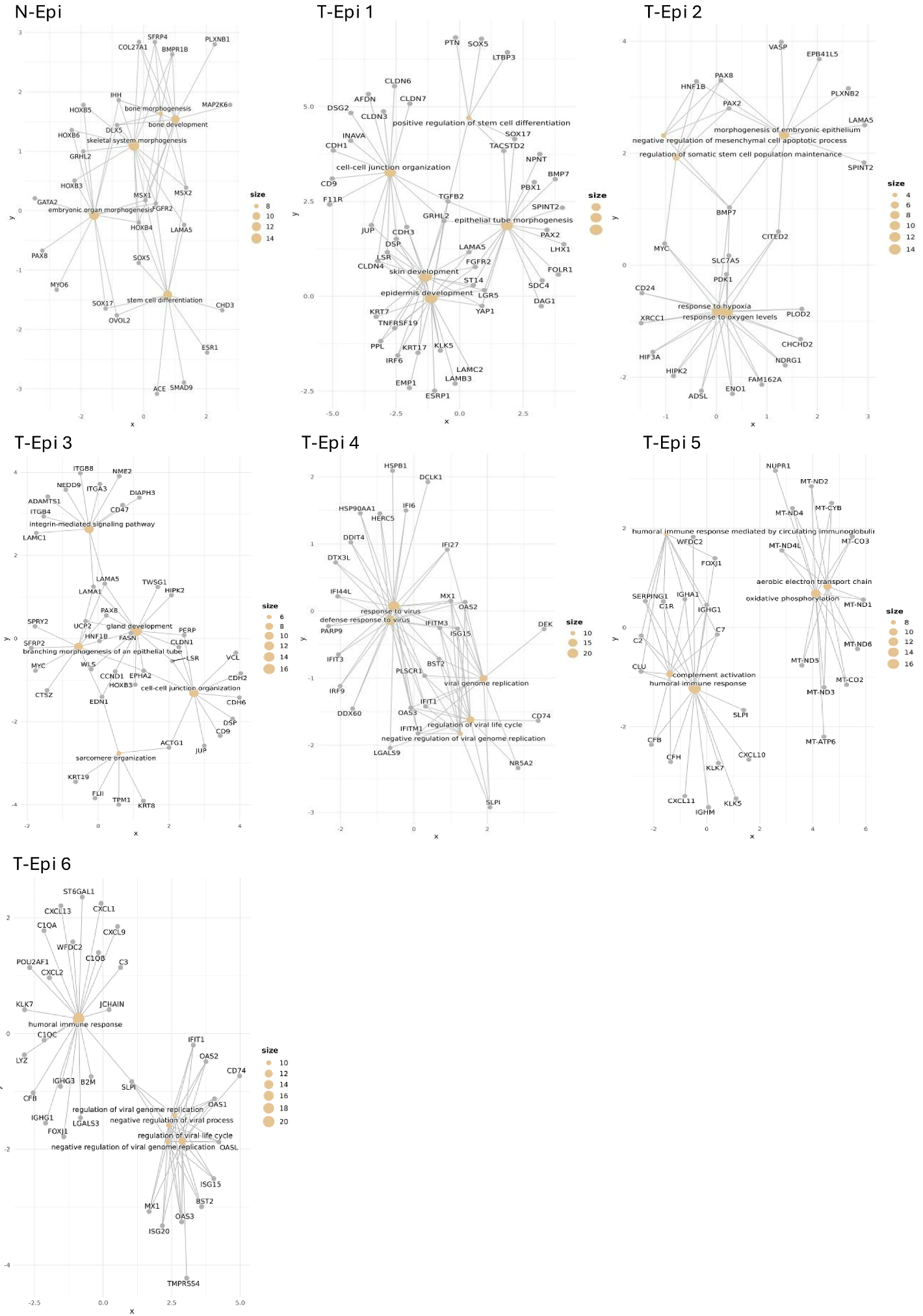
**

**Figure S3.** **Gene ontology network (cnet) plots of enriched Biological Process terms among upregulated genes in epithelial populations.** Dot size reflects the number of genes.


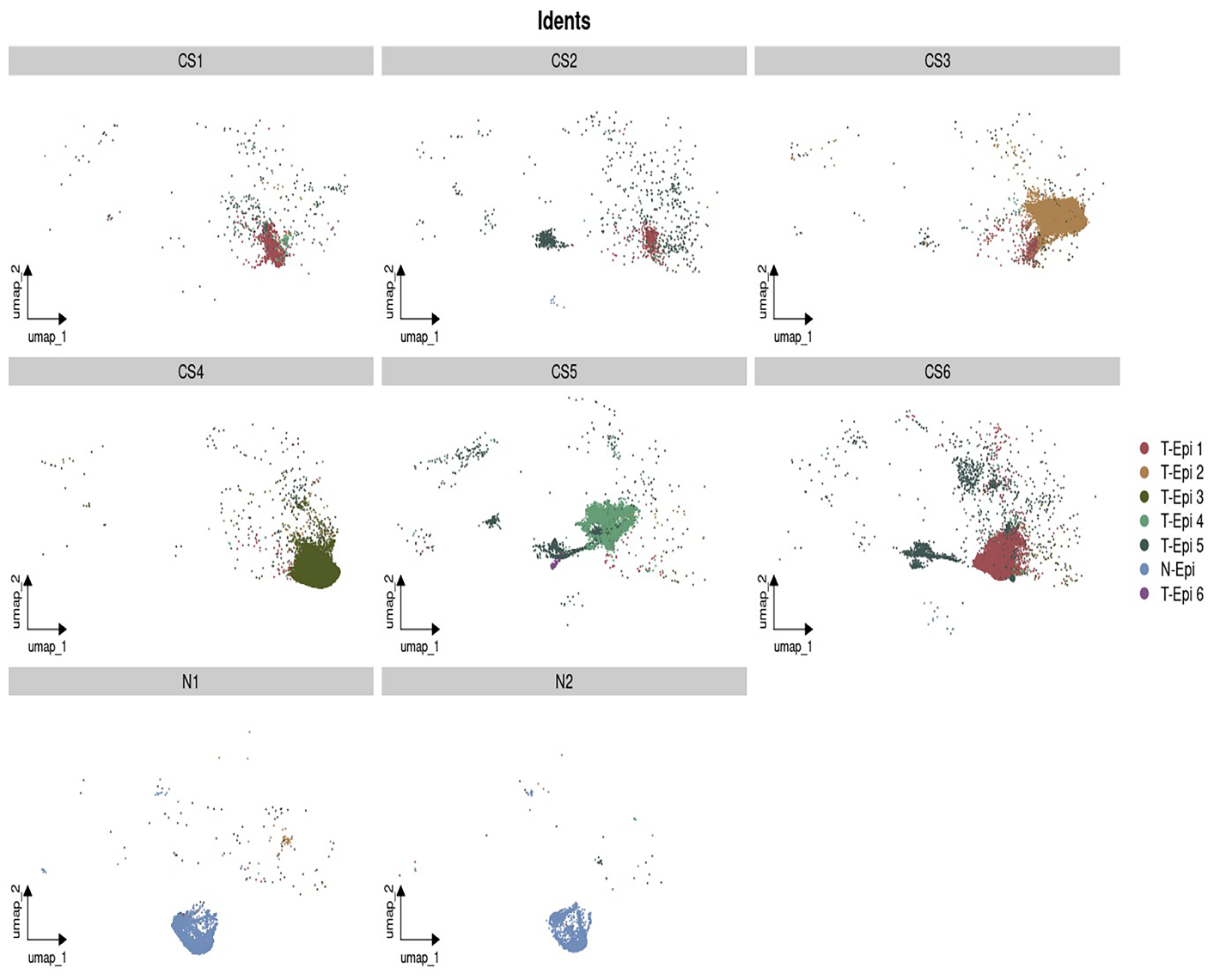


**Figure S4.** **UMAP visualization of epithelial clusters split by sample of origin.** Each panel shows the distribution of epithelial cell populations across individual tumors (CS1–CS6) and normal endometrium samples (N1, N2). While certain tumoral epithelial clusters (T-Epi 2, T-Epi 3, T-Epi 4, T-Epi 6) are restricted to specific tumors, clusters T-Epi 1 and T-Epi 5 are shared across multiple cases, although their relative abundance varies between samples.

**
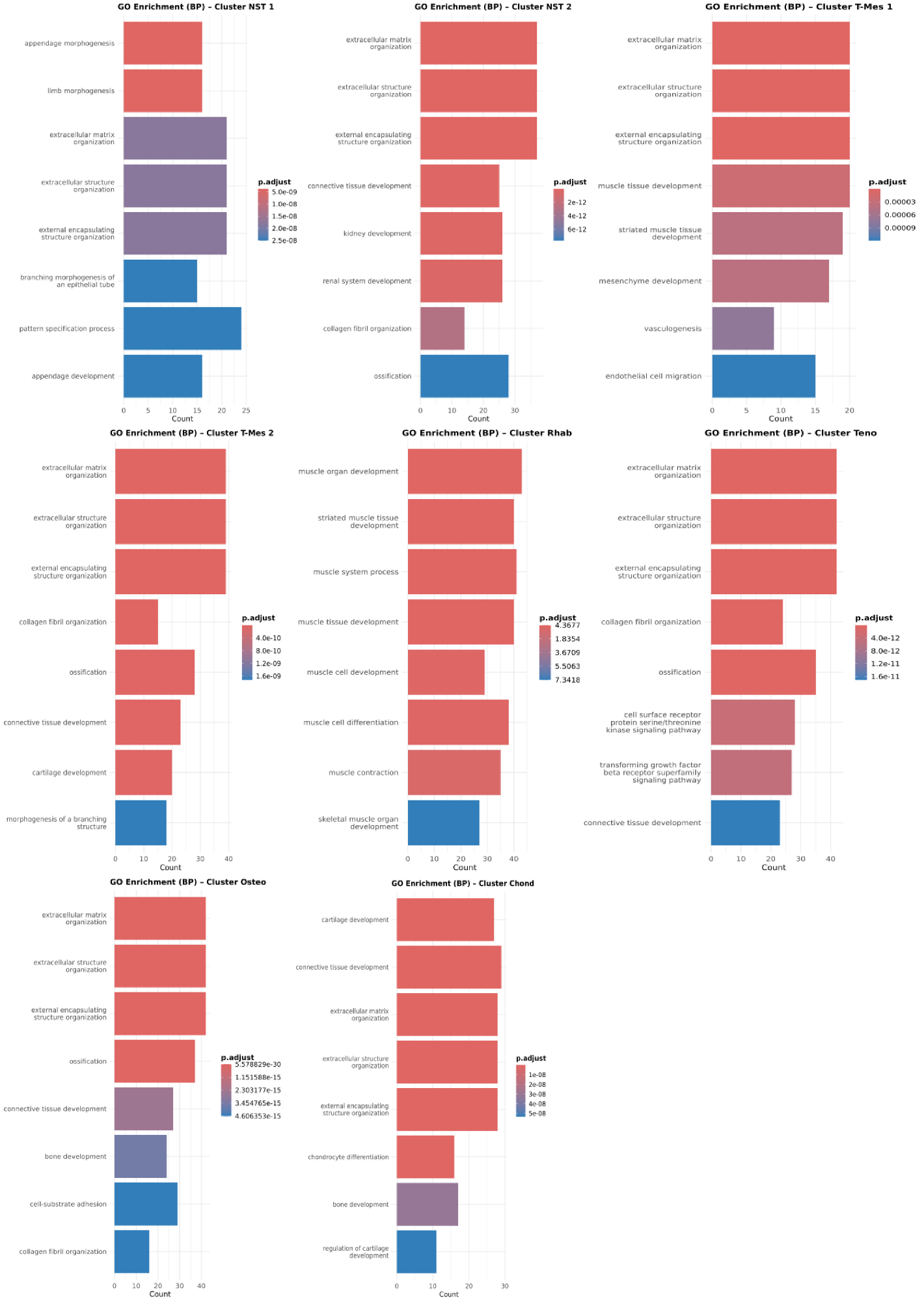
**

**Figure S5.** **Bar plots of enriched Biological Process GO terms among upregulated genes in mesenchymal populations.** Color scale represents statistical significance (adjusted *p* value < 0.05).

**
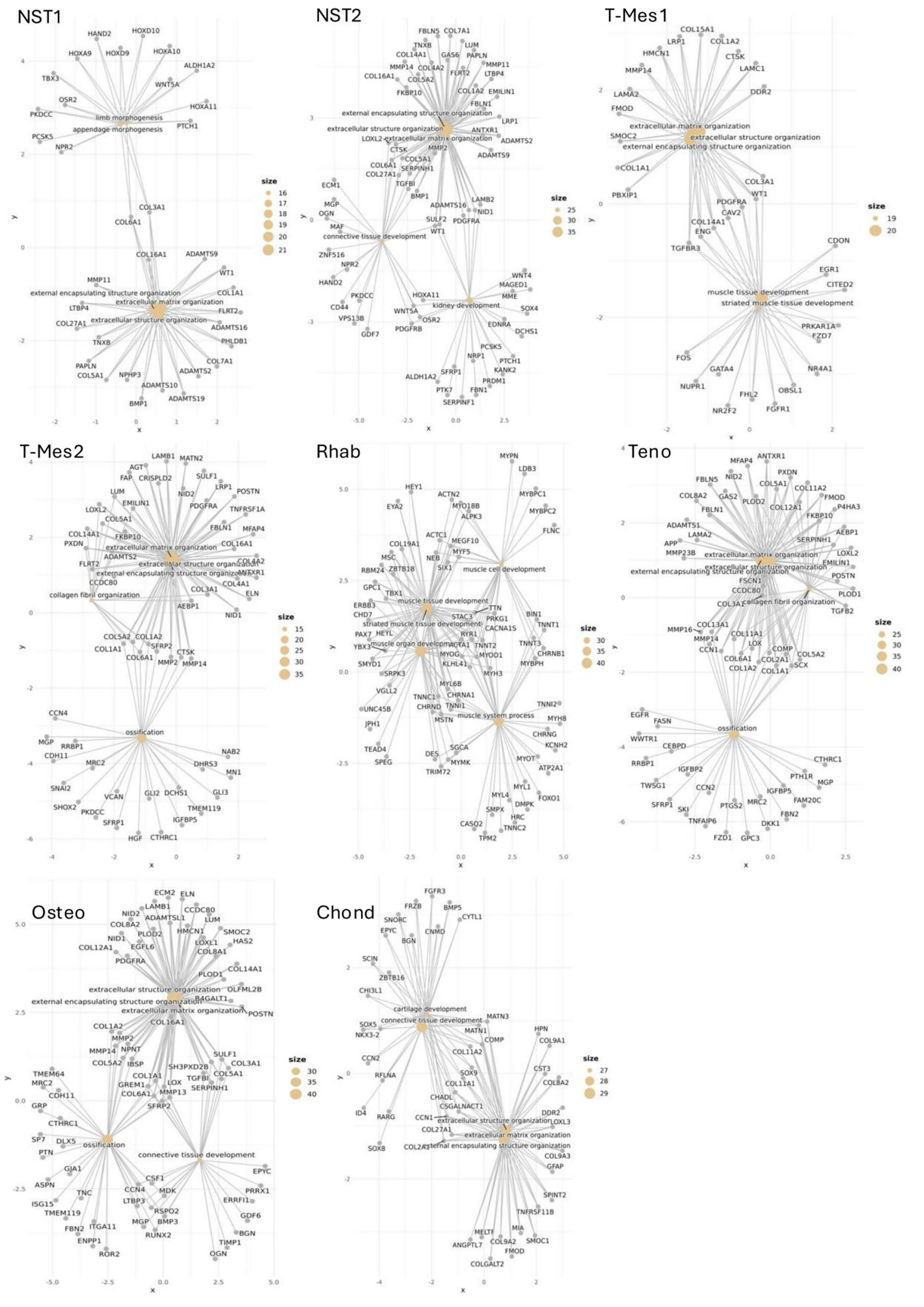
**

**Figure S6.** **Gene ontology network (cnet) plots of enriched Biological Process terms among upregulated genes in mesenchymal populations.** Dot size reflects the number of genes.

**
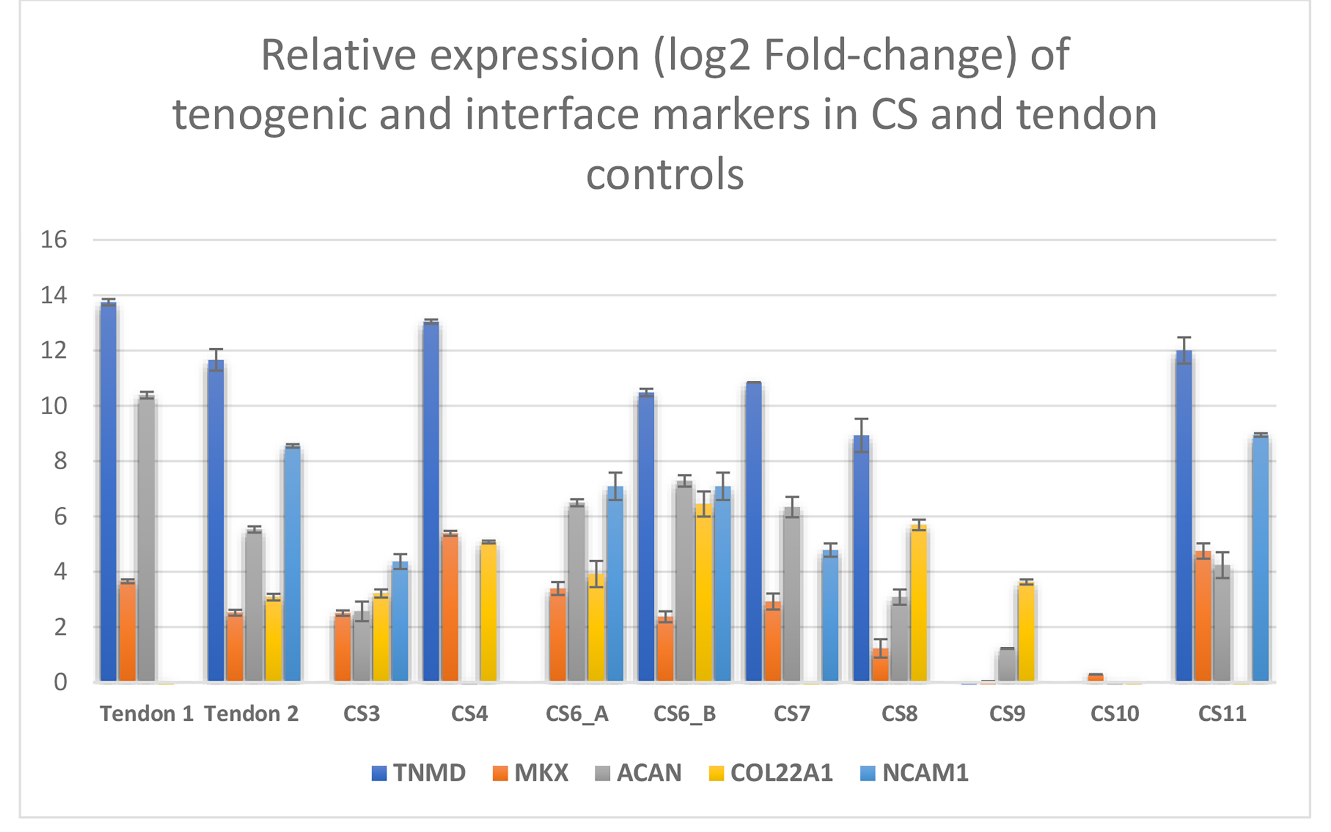
**

**Figure S7.** **Relative expression (log_2_ fold-change) of tenogenic (*TNMD*, *MKX*) and interface markers (*ACAN*, *COL22A1*, *NCAM1*) by RT-qPCR.** Gene expression was measured by RT-qPCR and calculated using the ΔΔCt method, with normal proliferative endometrium as a reference control. Bars represent mean ± SD from technical replicates. Only upregulated values (log_2_FC > 0) are shown; genes not detected (Ct > 35 in both sample and control) or downregulated relative to the control are not represented.


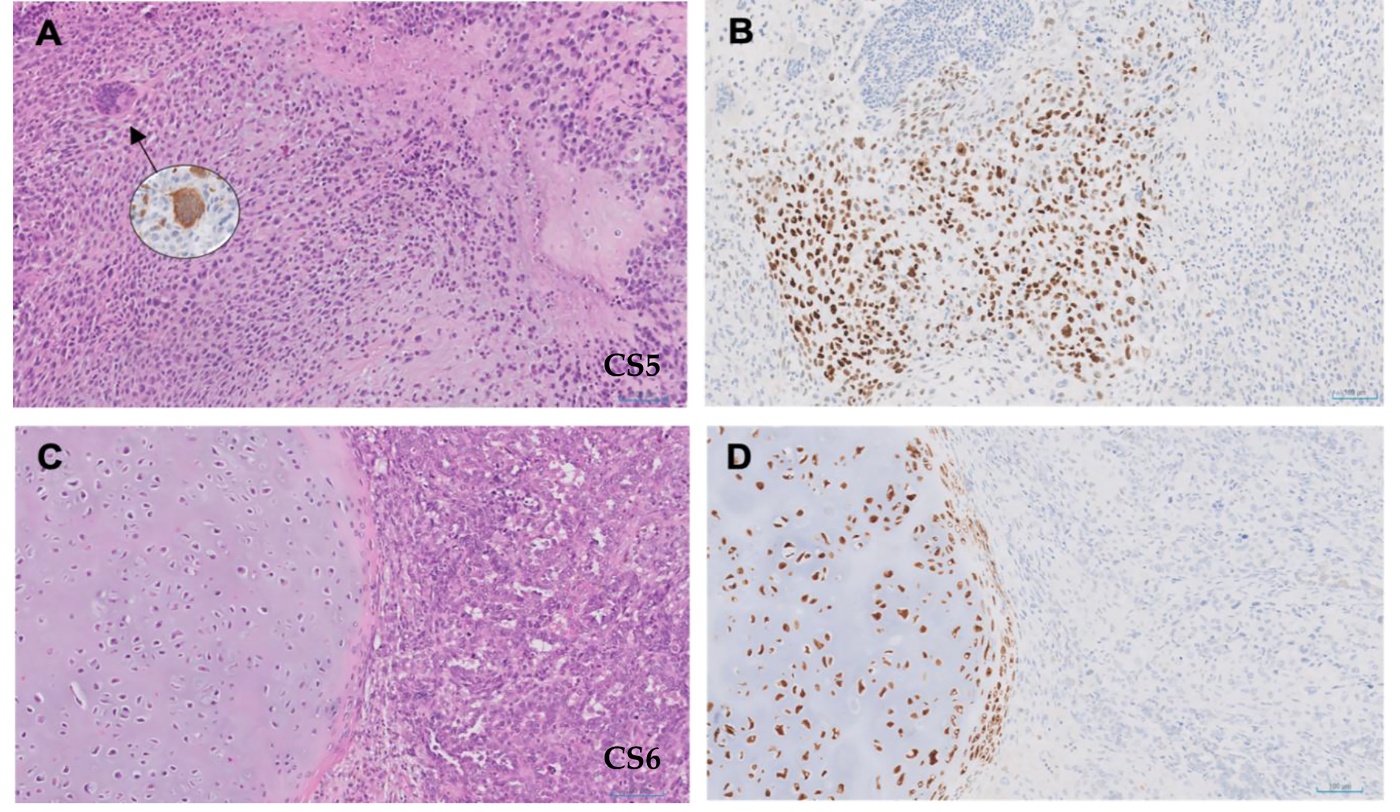


**Figure S8.** **Histological and immunophenotypic characterization of CS5 and CS6** (A) Haematoxylin and eosin (H&E) staining showing biphasic morphology in CS5. Giant multinucleated osteoclast-like cells are indicated by arrows, with the inset highlighting CD68 expression by immunohistochemistry (IHC). (B) IHC showing expression of SATB2 in CS5. (C) H&E staining showing biphasic morphology in CS6. (D) IHC showing expression of S100 in CS6. Scale bars: 100 μm.

**
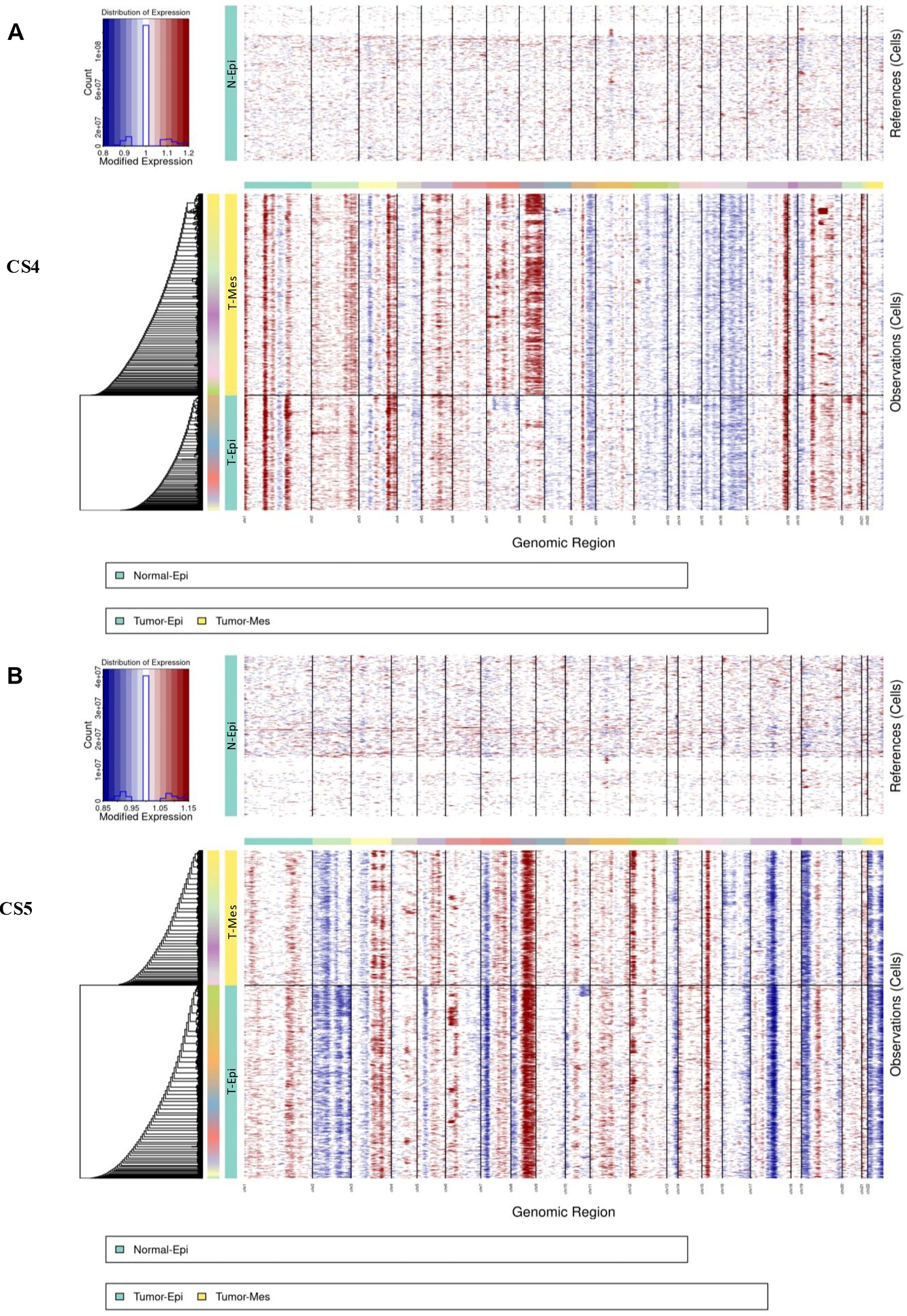
**

**Figure S9.** **Inferred copy number variation profiles of mesenchymal and epithelial cells in CS4 and CS5.** (A, B) Heatmaps showing expression-based copy number variation (CNV) inference for single cells from CS4 (A) and CS5 (B). Each panel compares tumor-derived epithelial and mesenchymal cells (T-Epi, T-Mes) against reference epithelial cells from normal endometrium (N-Epi). Red and blue signals indicate chromosomal gains and losses, respectively, across genomic regions (*x*-axis). Both cases display extensive CNV alterations in tumor compartments, with patient-specific patterns of chromosomal imbalance.

**
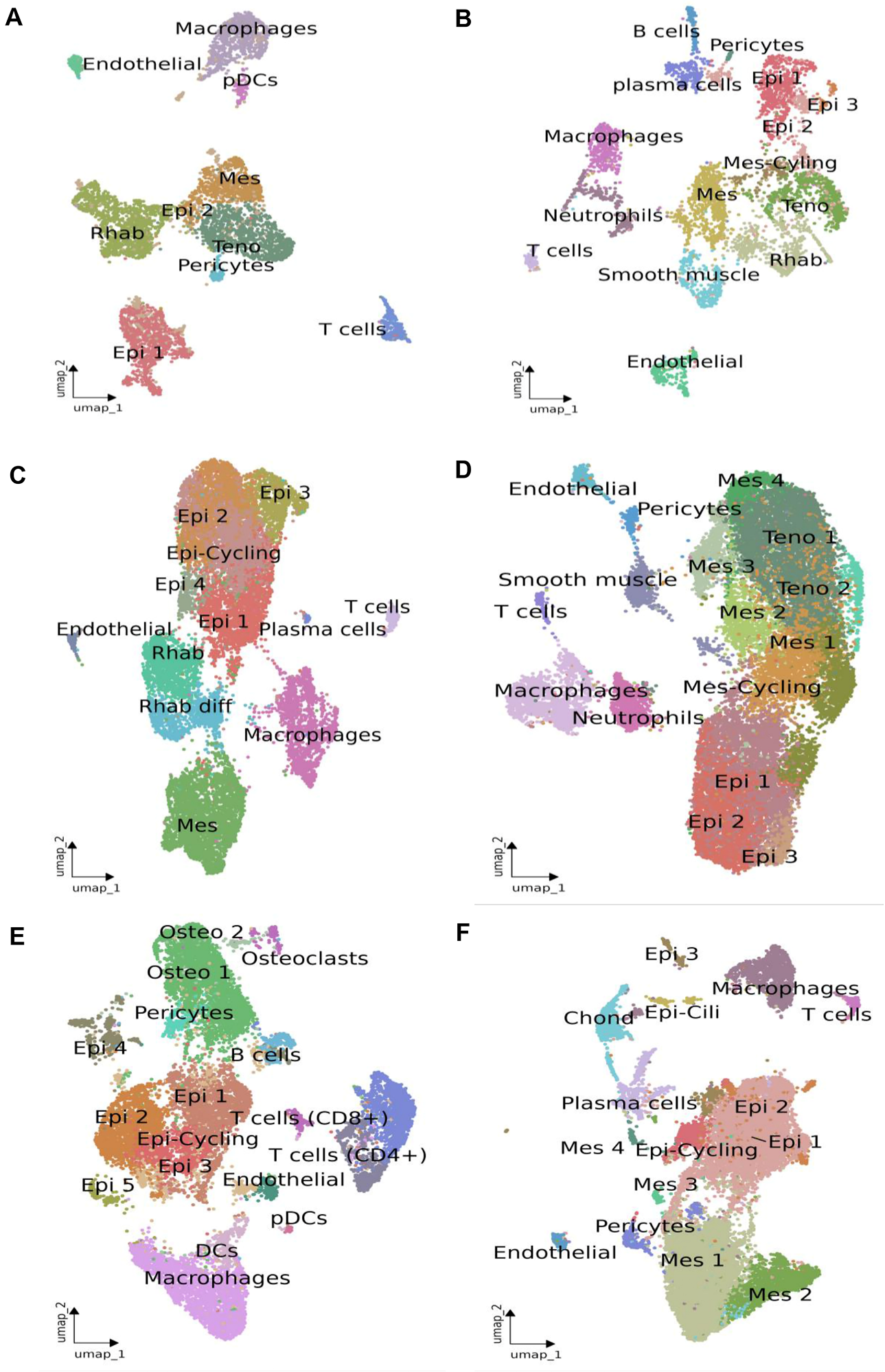
**

**Figure S10.** **UMAP plots of individual carcinosarcoma samples (CS1–CS6) showing tumor and microenvironment composition.** (A–F) UMAP representations of single-cell transcriptomic data from CS1 to CS6, respectively. Each plot shows the clustering of malignant epithelial (Epi), ciliated cells (Epi-Cili), malignant mesenchymal cells (Mes), rhabdomyoblasts (Rhab), differentiated rhabdomyoblasts (Rhab diff), tenoblasts (Teno), osteoblasts (Osteo), chondroblasts (Chond), proliferative subsets (Epi/Mes-Cycling), endothelial cells, plasma cells, macrophages, T cells, dendritic cells (DCs), plasmacytoid dendritic cells (pDCs), and neutrophils. The diversity of cell states and lineage trajectories highlights the inter-patient heterogeneity in both epithelial and mesenchymal compartments.
